# Mechanisms of microbial–neuronal interactions in pain and nociception

**DOI:** 10.1016/j.ynpai.2020.100056

**Published:** 2020-12-11

**Authors:** Valentina N. Lagomarsino, Aleksandar D. Kostic, Isaac M. Chiu

**Affiliations:** a Department of Immunology, Harvard Medical School, Boston, MA 02115, USA; b Joslin Diabetes Center, Boston, MA 02115, USA; c Department of Microbiology, Harvard Medical School, Boston, MA 02115, USA

**Keywords:** Sensory afferent neurons, Gut-extrinsic, Microorganisms, Pathogens, Symbionts, Pain, Visceral pain

## Abstract

Nociceptor sensory neurons innervate barrier tissues that are constantly exposed to microbial stimuli. During infection, pathogenic microorganisms can breach barrier surfaces and produce pain by directly activating nociceptors. Microorganisms that live in symbiotic relationships with their hosts, commensals and mutualists, have also been associated with pain, but the molecular mechanisms of how symbionts act on nociceptor neurons to modulate pain remain largely unknown. In this review, we will discuss the known molecular mechanisms of how microbes directly interact with sensory afferent neurons affecting nociception in the gut, skin and lungs. We will touch on how bacterial, viral and fungal pathogens signal to the host to inflict or suppress pain. We will also discuss recent studies examining how gut symbionts affect pain. Specifically, we will discuss how gut symbionts may interact with sensory afferent neurons either directly, through secretion of metabolites or neurotransmitters, or indirectly,through first signaling to epithelial cells or immune cells, to regulate visceral, neuropathic and inflammatory pain. While this area of research is still in its infancy, more mechanistic studies to examine microbial-sensory neuron crosstalk in nociception may allow us to develop new therapies for the treatment of acute and chronic pain.

## Highlights

• Molecular mechanisms of how microorganisms communicate with sensory afferent neurons.
• How pathogenic microorganisms directly communicate with nociceptor neurons to inflict pain on the host.
• Symbiotic bacterial communication with gut-extrinsic sensory afferent neurons.
• Plausible roles on how gut symbionts directly mediate pain and nociception.

## 1 Introduction

Pain is a distressing sensory experience, yet may be seen as evolutionarily beneficial to help animals adapt and avoid further exposure to noxious stimuli (Basbaum et al., 2009, Chiu, 2018, Dubin and Patapoutian, 2010, Lister et al., 2020). In the 1st century CE, the Greek philosopher, Celsus described pain (dolor) as one of the four cardinal signs of inflammation, along with warmth (calor), swelling (tumor) and redness (rubor) (*De Medicina*). The primary sensory afferent nerves responsible for responding to noxious stimuli and for mediating protective withdrawal reflexes were termed “nociceptors” by Sir Charles Sherrington in 1905. When nociceptors detect an injurious stimuli, they elicit an electrical signal that originates from their peripheral terminals to their cell bodies within the dorsal root ganglion (DRG), which sits close to the spinal cord, and then signal via their central terminals to the spinal cord and the brain to be processed as the sensation of pain (Basbaum et al., 2009). In the 17th century, René Descartes was one of the first philosophers to describe this somatosensory experience as a signaling cascade (Benini and DeLeo, 1999). Centuries later, we have a better understanding of the neural networks underlying pain (Moayedi and Davis, 2013), but still do not have sustainable therapies for many debilitating chronic pain-associated diseases. Only within the past few decades has there been an appreciation for cellular players outside the nervous system, such as immune cells and microbes, playing key roles in pain (Defaye et al., 2020) ).

The human body is home to trillions of microorganisms that colonize us at birth and are distinct to each of us. The body is also exposed to potential microbial threats including pathogens that invade barrier tissues including the gut, skin and lungs. Peripheral sensory neurons including nociceptors densely innervate these barrier sites and are the body’s way of interfacing with the environment. Distinct sensory afferent neurons recognize mechanical stretch, chemical, and environmental stimuli, and initiate a signaling cascade of electrical impulses to the central nervous system (CNS) to mediate pain, itch, touch, and proprioception (Abraira and Ginty, 2013, Basbaum et al., 2009, Chiu, 2018, Dubin and Patapoutian, 2010). A better fundamental understanding of how microbes, both symbiotic and pathogenic, communicate with sensory neurons to elicit these signaling cascades could lead to new insights into pain as well as other sensory modalities.

Recent work has shown that like immune cells, nociceptors are able to directly detect microbial products. There has been extensive work showing that pathogenic microorganisms are capable of directly and indirectly communicating with nociceptors at different barrier sites to mediate pain behavior, which then induces neurogenic inflammation and neuro-immune circuits that regulate host defense (Baral et al., 2019, Chiu et al., 2012). Some pathogens have been found to directly activate nociceptors during an infection leading to the release of neuropeptides which can then signal to activate the innate and adaptive immune systems (Baral et al., 2019, Pavlov and Tracey, 2017). Bidirectional signaling between the nervous and immune system during an infection is critical for the health of the host but can be exploited by pathogens for survival (Pinho-Ribeiro et al., 2017, Pinho-Ribeiro et al., 2018). By contrast with pathogens, the role of specific commensal and mutualistic microbes and the microbiome in direct signaling to sensory afferents in pain is less well understood. Given the need for better therapies for the management of pain, a better fundamental understanding of the interactions between symbiotic microbes and the nervous system is needed.

In this review, we aim to describe the current state of knowledge of how both pathogenic microbes and resident commensal and mutualist microbes affect nociceptors and the perception of pain. We will first focus on the known molecular mechanisms through which pathogenic microbes communicate with sensory afferent neurons to induce pain behavior. We will discuss how bacterial pathogens use toxins, *N*-formyl peptides, and other molecular patterns to cause pain. Further, we will discuss how fungal and viral pathogens affect pain. Then, we will highlight evidence suggesting that gut symbionts may also contribute to onset or attenuation of pain and suggest potential molecular pathways through which they may be interacting with sensory afferents to mediate this behavior. We will discuss recent work outlining potential mechanisms through which symbionts influence many forms of pain, including inflammatory pain, visceral pain, and neuropathic pain. Further, we will discuss signaling from microbially-produced metabolites and products that directly bind to neurons, as well as discuss indirect mechanisms through which gut microbes signal to immune cells and epithelial cells, which coordinate signals to neurons. Herein, we will primarily focus on primary afferent nociceptors that reside within the dorsal root ganglia (DRG) and how they interact with microbial stimuli. We may refer to these neurons by the type of receptor they express such as TRPA1, TRPV1+, or Nav1.8+ (Julius, 2013, Patapoutian et al., 2009). The goal of this review is to give an overview of the different modes of communication that microorganisms can have with sensory afferent neurons in nociception.

### 1.1 Stratifying the role of the microbiome in pain: commensals and mutualists versus pathogens

Hosts and their microbiome have a longstanding evolutionary relationship that is important for the development and health of the host (Foster et al., 2017b, McFall-Ngai, 2014). In humans, microorganisms such as bacteria, viruses, fungi and parasites have been found to affect the nervous system and influence a range of complex behaviors such as cognition, stress response, nociception, social learning, and feeding behavior (Cryan and Dinan, 2012). Symbiotic microbes live in ecosystems (Costello et al., 2009) that colonize the oral and nasal cavities (Wade, 2013), airways (Kumpitsch et al., 2019), skin (Schommer and Gallo, 2013), genitourinary tract (Ma et al., 2012) and the gut (The Human Microbiome Project Consortium, 2012). Microbes have been found to reside in close proximity to peripheral sensory neurons at these barrier sites, including the gut (Grundy et al., 2002), which allows potential crosstalk between neurons and microbes.

Early work to understand gut–nervous system crosstalk occurred in the context of feeding behavior. In 1902, Nobel laureate Ivan Pavlov illustrated the first gut-brain connection through his experiments on eating behavior and the release of gastric juices by digestive glands in the stomach. Through performing vagotomy experiments, he found that the sole act of eating leads to vagal nerve-mediated gastric changes in the gut, even when the food is not physically reaching the stomach. Today, we know that microbes have the ability to synthesize and regulate neurotransmitters including γ-aminobutyric acid (GABA) and serotonin, as well as produce their own metabolites and products that are capable of modulating feeding behavior (De Vadder and Kovatcheva-Datchary, 2014). Interestingly, evolutionary biologists have proposed that the enteric nervous system, a gut-intrinsic sensory nervous system, may have evolved before the CNS and the anatomy of this system is highly conserved across animals (Furness and Stebbing, 2018). This discovery raises many questions as to why microbiota communicate with the nervous system, and how they mediate signals along a gut-brain neural circuit to control a range of behaviors, including pain.

With the advancement of germ-free (GF) animals and computational microbiology, the gut microbiome has been found to modulate a range of host neurological functions and behaviors (Cryan et al., 2019). It has been demonstrated that GF mice, which live in the complete absence of live microorganisms, exhibit a range of neurodevelopmental abnormalities that can be abrogated upon colonization with a complex microbiome (Sharon et al., 2016, Vuong et al., 2017, Hsiao et al., 2013). Further, the gut microbiome has been implicated in affecting a range of behaviors such as social learning (Gilbert, 2015, Hsiao et al., 2013), fear conditioning (Hoban et al., 2018, Chu et al., 2019), emotional behavior (Bravo et al., 2011, Zheng et al., 2016), and stress related behaviors such as nociception and anxiety (Foster et al., 2017a). Microbes have been found to influence blood brain barrier permeability through regulating expression of tight junction proteins in the endothelium (Braniste et al., 2014). It has also been shown that microbes and microbial metabolites can drive microglia activation and neuroinflammation (Dopkins et al., 2018) and this may underlie neurodegenerative diseases like Parkinson’s disease (Sampson et al., 2016) and Alzheimer’s disease (Minter et al., 2016). However, the exact molecular and cellular underpinnings through which microbes influence neuronal function are still largely unclear and are a major topic of research interest.

Pain behaviors have also been found to be regulated significantly by the gut microbiome. Germ-free (GF) mice displayed reduced mechanical allodynia after intraplantar injections of carrageenan and LPS and reduced acute nociceptive responses to formalin injection compared to conventional mice (Amaral et al., 2008). The reduced sensitivity correlated with decreased local levels of TNF-α, IL-1β, or CXCL1 in carrageenan-injected footpads. In this study, the presence of commensal microbes cause decreased anti-inflammatory IL-10 signaling, potentially in a Toll-like receptor (TLR)-dependent manner (Amaral et al., 2008). TLRs are molecular sensors of the innate immune system that recognize microbial and damage-associated molecular patterns, and are commonly found on immune cells including macrophages and dendritic cells (Rakoff-Nahoum and Medzhitov, 2008). Some TLRs have also been found to be expressed on neurons (Liu et al., 2012b). We will discuss the role of TLRs in pain in more detail later in the review article. Chemotherapy induced neuropathic pain is a major cause of chronic pain and suffering in cancer patients (Costigan et al., 2009). Recent work showed that germ-free mice were protected from development of mechanical hyperalgesia following administration of a chemotherapy drug, oxaliplatin (Shen, 2017). LPS from the gut microbiome was thought to reach immune cells such as macrophages in the DRG to induce TLR4 signaling and drive mechanical hypersensitivity. These studies indicate that the gut microbiome contributes to mediating pain in inflammatory and neuropathic pain sensitization. In a study of visceral pain in mice using measurements of visceromotor responses (VMR induced by a colorectal distension), germ-free mice had increased baseline sensitivity compared to conventionally colonized mice, indicating that endogenous microflora suppress baseline sensitivity (Luczynski et al., 2017). This correlated with morphological changes in the anterior cingulate cortex and the periaqueductal gray (PAG), suggesting that the microbiome influences neuronal circuitry in brain regions involved in visceral pain processing. Despite these studies, the specific underlying molecular and cellular mechanisms by which commensal gut or skin microbes signal to nociceptor neurons, spinal cord or brain circuits to regulate neuronal function are largely undefined. The overall role of the microbiome in regulating distinct chronic inflammatory and neuropathic pain conditions also remain to be determined. A recent study showed that different gut commensal microbes are capable of inducing DRG nociceptor calcium influx and action potential firing *in vitro*, suggesting that commensal microbes are able to directly signal to nociceptor neurons to modulate neural activity; however, the underlying molecular mechanisms are still undefined (Yissachar et al., 2017). It would be important to determine in the future how specific members of the microbiome interact with each other to synthesize and contribute specific molecules that in turn may signal to nociceptor neurons. Furthermore, the role of dysregulated microbiome in leading to imbalanced metabolic or microbial products that could heighten or mediate chronic pain remains to be determined.

Pathogenic infections of barrier sites, including the skin, airways and gut, are often accompanied by pain. The molecular mechanisms of how these pathogenic microorganisms cause pain are more well understood. A major distinction of pathogens from other microbiome constituents is that pathogens are defined as having a net-detrimental relationship with the host, generally causing damage to host tissues, rather than a commensal or mutualistic relationship (Bullman et al., 2017). Symbionts, commensals and mutualists, can become pathogenic in situations of dysbiosis, where blooms of specific pathobionts can lead to significant tissue inflammation and pathology in the gut, skin and other barriers, resulting in pain. Some microorganisms, such as the fungus *Candida albicans*, are opportunistic pathogens, which means that at homeostasis they have a commensal relationship with their host but upon certain conditions, such as stress or dysbiosis, can become pathogenic and have a net negative affect on their host. Nociceptor sensory neurons may warn against the presence of pathogens and be the first line of defense. Extensive work has shown that nociceptors are able to directly detect bacterial, fungal and viral pathogens that have pathogenic molecular patterns, as well as other molecular patterns, which we will discuss thoroughly in the next few sections. Neurons also actively communicate with immune cells, such as macrophages, T cells and mast cells, at barrier cites in a bidirectional fashion (Baral et al., 2019). In some cases, recognition of pathogenic molecules or by immune cell mediators facilitates neurogenic inflammation that drive host protection against the pathogens, and in other cases pathogens directly suppress sensory neuronal activation or immune cell activation to facilitate their survival or transmission (Pinho-Ribeiro et al., 2018). In the following section, we will focus on the known and potential molecular mechanisms by which pathogenic microorganisms act on nociceptors.

### 1.2 Molecular mechanisms of pain: how pathogens act on nociceptive neurons

**Fig. 1:**
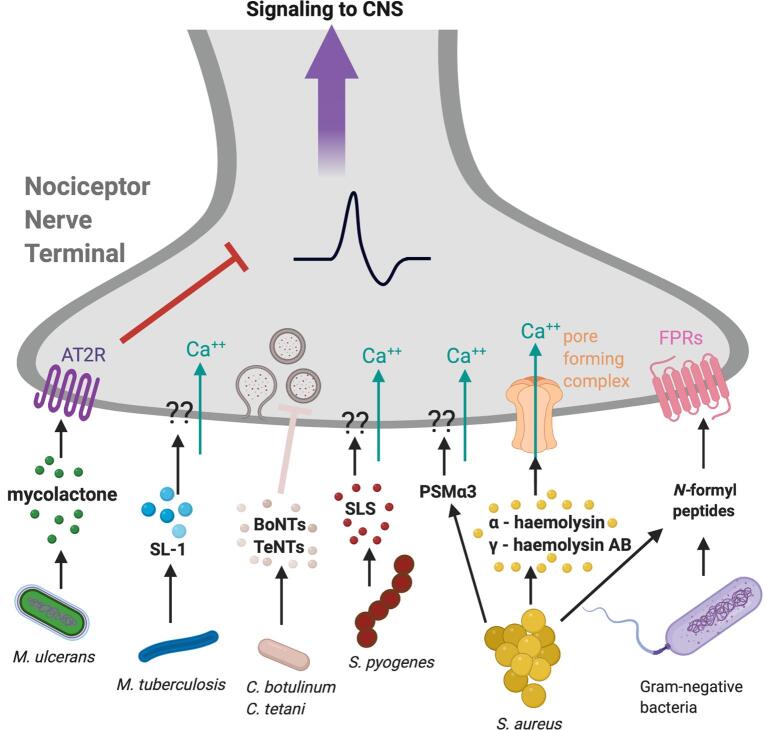
**Bacterial toxins and*****N*****-formyl peptides that modulate nociception.***Mycobacterium ulcerans*, a pathogen that leads to the Buruli ulcer, decreases pain sensation by secreting mycolactone which binds to angiotensin II receptor (AT2R) which leads to an increase of potassium to hyperpolarize nociceptors. *Mycobacterium tuberculosis* (Mtb), a pathogen that causes pulmonary tuberculosis (TB) activates nociceptors by secretion of sulfolipid-1 (SL-1). SL-1 increases calcium influx in these neurons to cause induce. *Clostridium botulinum*, *Clostridium tetani* secrete toxins such as botulinum neurotoxin (BoNT) and tetanus neurotoxin (TeNT) that block neurotransmission by cleaving SNARE proteins in neurons which prevents synaptic vesicles containing neurotransmitters from binding to synaptic cleft for release. *Streptococcus pyogenes*, a pathogen that causes necrotizing fasciitis, causes pain by release of the pore-forming peptide toxin streptolysin S (SLS), which leads to an influx of calcium. *Staphylococcus aureus*, a pathogen that leads to boils and painful rashes, activates neurons by release of 3 major classes of pore-forming toxins (PFTs) including α-haemolysin, phenol soluble modulin α3 (PSMα3) and γ-haemolysin AB. *S. aureus* and other gram-negative bacteria also secrete *N*-formyl peptides which can bind to formyl peptide receptors which can activate nociceptors to induce pain. Image created with BioRender.com.

Bacterial, fungal and viral infections have all been found to be associated with pain. Pathogens cause tissue damage through secreted virulence factors including pore-forming toxins and other mediators that modulate host immune cell signaling. As part of the immune response, host cells express pathogen recognition receptors (PRR) that can detect pathogen associated molecular patterns (PAMPs). There is evidence that toxins and PAMPs can act directly on sensory afferents to produce pain through involvement of formyl peptide receptors (FPR), nociceptive transient receptor potentials (TRP) channels, and TLRs (Chiu, 2018). There is also evidence that pathogens can cause pain indirectly, by first damaging host tissues. This can then cause the release of endogenous signals, alarmins or danger associated molecular patterns (DAMPs), that can activate PRRs such as TLRs on neurons and immune cells. In this section, we will outline mechanisms of how pathogenic bacteria directly interact with sensory afferents through secretion of pore-forming toxins, N-formyl peptides and other secreted molecules (Fig. 1). Further, we will discuss the mechanisms of how microbial pathogens such as bacteria, fungi and viruses have different molecular patterns that interact with sensory afferents to cause pain.

#### 1.2.1 Bacterial infections, pore-forming toxins, and activation of pain

*Staphylococcus aureus* is a major bacterial pathogen that causes painful abscesses and boils. One of the first studies showing that bacteria directly induced neuronal activation came from the observation that mechanical and thermal hyperalgesia during *S. aureus* infection correlated with bacterial expansion rather than tissue swelling or immune cell influx (Chiu et al., 2013). *S. aureus* and several other bacterial pathogens were found to directly induce calcium influx in DRG nociceptor neurons.

One of the key mechanisms by which *S. aureus* induces pain during infection is through secretion of the pore-forming toxin (PFTs) α*-hemolysin* (Hla). Hla is a pore forming toxin that directly binds to A disintegrin and metalloproteinase domain–containing protein 10 (ADAM10) on neurons and other cells. In a nociceptive nerve terminal, this leads to cation influx and neuronal depolarization, producing action potential firing and pain perception. Hla was necessary and sufficient to produce pain during infection. It was found that absence of α*-hemolysin* greatly diminished both acute nocifensive pain following infection and hyperalgesia following infection (Chiu et al., 2013, Blake et al., 2018). *S. aureus* produces 3 major classes of pore-forming toxins (PFTs) including hemolysins, leukocidins, and phenol soluble modulins (PSMs). It was further found that in addition to Hla, both PSMs and the bicomponent leucocidin HlgAB can also directly activate nociceptor neurons to produce action potential firing in culture and pain production *in vivo* (Blake et al., 2018). Finally, it was found that a positively charged form of lidocaine, QX-314, is able to enter into nociceptor neurons, likely due to pore formation, and potently silence pain during *S. aureus* infection (Blake et al., 2018).

PFTs are key virulence factors for several classes of bacterial pathogens, facilitating their spread and survival in hosts by damaging host cell membranes and acquisition of nutrients. It may make physiological sense, therefore, that these highly damaging toxins could be directly detected by nociceptive neurons. The role of PFTs in pathogen-induced pain was further extended to another major skin and soft tissue-invading pathogen, *Streptococcus pyogenes* (Pinho-Ribeiro et al., 2018).

*S. pyogenes* is a gram-positive pathogen that is the leading cause of necrotizing fasciitis (flesh-eating disease), a life-threatening infection characterized by severe “pain out of proportion” with other symptoms at early stages of infection. In a mouse model of *S. pyogenes*-induced necrotizing fasciitis, it was found that the Streptococcal PFT streptolysin S (SLS) is a critical mediator of both acute nocifensive pain and mechanical hyperalgesia (Pinho-Ribeiro et al., 2018). SLS is thought to bind non-specifically to cell membranes and induce cation influx. In culture, SLS was critical for the induction of calcium influx into TRPV1 + neurons and this was abolished in bacteria lacking SLS production. *In vivo*, pain was absent following infection of mice by M1 and M3 strains of *S. pyogenes* deficient in *sagA*, the gene encoding SLS. Furthermore, a neutralizing antibody against SLS significantly attenuated pain caused by *S. pyogenes* infection. Interestingly, downstream of nociceptor activation, local release of the neuropeptide calcitonin-gene related peptide (CGRP) led to suppression of neutrophil recruitment and killing of bacteria. Therefore, *S. pyogenes* produces pain through SLS and subsequently hijacks a nociceptor-driven immunosuppression to facilitate its survival during infection (Pinho-Ribeiro et al., 2018).

#### 1.2.2 N-Formyl peptides and pain

Bacteria express *N*-formylases which leads to the production of *N*-formyl peptides. Mammals have evolved formyl peptide receptors (FPRs) which are 7 transmembrane G protein–coupled receptor (GPCR) that can induce chemoattraction in phagocytic leukocytes (Yang and Chiu, 2017). FPR1 is expressed by nociceptive sensory neurons, and it was found that FPR1-/- mice had decreased mechanical allodynia in mice following *S. aureus* injection (Chiu et al., 2013). *N*-formylated peptides fMLF from *E. coli* and fMIFL from *S. aureus* also produce mechanical allodynia when injected into mice. Given that formyl peptides are a metabolic signature of most bacteria, it could be one way that mechanical allodynia is induced during nociception.

#### 1.2.3 *M. tuberculosis* and cough

Cough is a nociceptive reflex driven by vagal sensory neurons that innervate the lungs. Though it is distinct from pain, there are important analogous functions for host protection. *Mycobacterium tuberculosis* (Mtb) is a pathogenic bacterium that is the causative agent for pulmonary tuberculosis (TB). The primary symptom of TB is cough, which is also one of the main ways in which the disease is transmitted. Recently, it was shown that the secreted bacterial sulfolipid-1 (SL-1) was the critical bioactive molecule that directly activates sensory neurons to induce cough (Ruhl et al., 2020). Vagal and DRG TRPV1+ neurons exposed to Mtb and its extract *in vitro* had a rapid increase in intracellular calcium, and this was greatly diminished in isogenic strains of MTb lacking genes for the synthesis of the sulfolipid. Further, they show that SL-1 was necessary and sufficient to induce cough in a guinea pig model of TB infection (Ruhl et al., 2020). This study provides novel insight into the role of the somatosensory nervous system in respiratory infections and opens up a new direction for studying these illnesses.

#### 1.2.4 Bacterial toxins that inhibit neurotransmission and pain

*Clostridium botulinum*, *Clostridium tetani* and other related family members of bacteria have evolved toxins including botulinum neurotoxin (BoNT) and tetanus neurotoxin (TeNT) that specifically act as neurotoxins to block neurotransmission. BoNTs and TeNTs block neurotransmission by cleaving SNARE proteins in neurons, which prevents synaptic vesicles containing neurotransmitters from binding to synaptic cleft for release. BoNTs consist of A-G serotypes, which each target distinct SNARE proteins in cells, including SNAP25 and VAMP1. Currently BoNT/A is widely used for dermatological applications because of its long-lasting nature of blocking motor neuron activity. BoNT/A is also able to target nociceptor neurons in addition to motor and sympathetic neurons, and there is interest in applying it in the treatment of pain and in particular chronic migraine. TeNT is produced by *C. tetani* and has been proposed to block neurotransmission by binding to two independent receptors on neurons, polysialogangliosides (PSGs) and proteinaceous receptors. An extensive review on the molecular mechanisms through which pathogenic metabolites and bacterial toxins including BoNTs directly interact with neurons can be found from Yang and Chiu (2017).

*Mycobacterium ulcerans* is a bacterial pathogen that causes large painless skin lesions called Buruli ulcers. *M. ulcerans* was found to secrete a mycolactone which takes advantage of the angiotensin pathway in neurons to modulate pain. Mycolactone acts as an analgesic through binding to a type 2 angiotensin II receptor (AT2R) leading to nociceptor hyperpolarization through potassium influx through TRAAK channels (Marion et al., 2014). Future identification of other bacterial toxins or molecular mediators that could silence pain is an exciting area of research, as they could potentially be repurposed for the treatment of acute or chronic pain.

#### 1.2.5 Bacterial PAMPs and pain

**Fig. 2:**
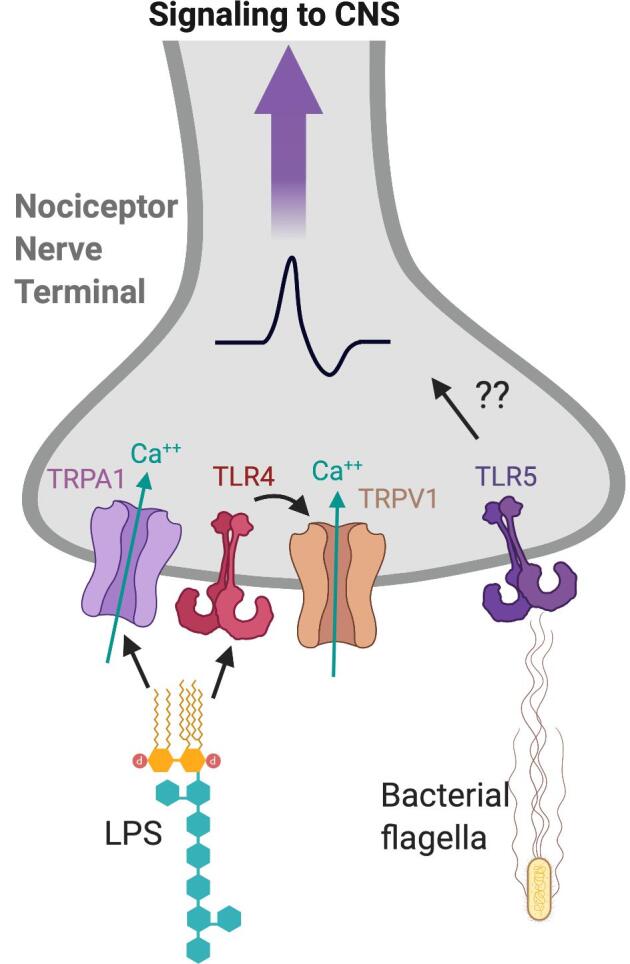
**Bacterial PAMPs that cause nociception.** Cell wall components of Gram-negative bacteria, including Lipopolysaccharide (LPS), can activates nociceptors through either neuronal TLR4 or direct signaling to the transient receptor potential cation channel subfamily A member 1 (TRPA1) ion channel. Gram-negative bacteria can also activate nociceptors through binding of bacterial flagellin to TLR5, however it is unclear how that leads to neuronal activation. Image created with BioRender.com.

Some common bacterial PAMPs include lipopolysaccharide (LPS), flagellin, and peptidoglycans. Nociceptors have been found to express pathogen recognition receptors (PRRs) such as TLR4, TLR5, and TLR7 (Liu et al., 2012a, Liu et al., 2012b). It has also been found that TLR4 and TLR7 colocalize with TRPV1 in trigeminal and DRG neurons (Helley et al., 2015) (Fig. 2).

LPS is a bacterial endotoxin that is found on the outer cell wall of all Gram-negative bacteria. Canonically, LPS binds to TLR4, CD14 and MD2 which leads to MyD88-induced NF-κB/IRF/MAPK signaling. This signaling cascade can induce an inflammatory response and activation of the adaptive immune system. Trigeminal nociceptor neurons express TLR4 or receptors for CD14, which allows them to directly respond to bacterial LPS (Wadachi and Hargreaves, 2006). Further, LPS was found to activate mouse colonic innervating DRG neurons independently of TLR4 (Ochoa-Cortes et al., 2010). It was later shown that LPS could increase neuronal excitability through mediating the gating of the TRPA1 ion channel on nociceptors (Meseguer et al., 2014). Together, these studies show that LPS can activate nociceptors through TLR4 and non-canonical signaling pathways of TRPA1 to cause pain and other symptoms such as nausea and fever.

Bacterial flagella are used by some bacteria for locomotion. TLR5 is a major host innate immune receptor that recognizes bacterial flagellin for host defense (Hayashi et al., 2001). Recently, TLR5 was found specifically on a subset of A-fiber sensory neurons (Xu et al., 2015). Concurrent application of flagellin, the TLR5 ligand, with the positively charged lidocaine derivative QX-314 was able to block sensitization of these neurons in a dose dependent manner. However, administration of QX-314 to Tlr5-/- mice did not block sensitization, which suggests that TLR5 is coupled to a separate transporter that allows the entry of membrane impermeable QX-314 (Xu et al., 2015). Furthermore, this administration greatly diminished neuropathic pain caused in mouse models of chemotherapy, nerve injury and diabetic neuropathic pain.

Further, one paper showed that stimulating DRG neurons with TLR3/7/9 ligands increases the expression of TLR3/7/9 receptors, suggesting that these receptors may play a role in mediating an inflammatory response and pain hypersensitivity (Qi et al., 2011). They also showed that suppression of TLR9 lead to reduced pain sensitivity to heat in a neuropathic pain model (Qi et al., 2011). Given the molecular heterogeneity of DRGs and recent single cell classification of nociceptor neurons (Hu et al., 2016.; Sharma et al., 2020), we may gain better insight into what TLRs and other pathogenic recognition receptors are expressed by different sensory afferent DRG neurons through deeper analysis of distinct neuronal subsets and their future functional characterization in response to bacterial ligands Figure 2.

#### 1.2.6 Viral pathogens and pain

**Fig 3:**
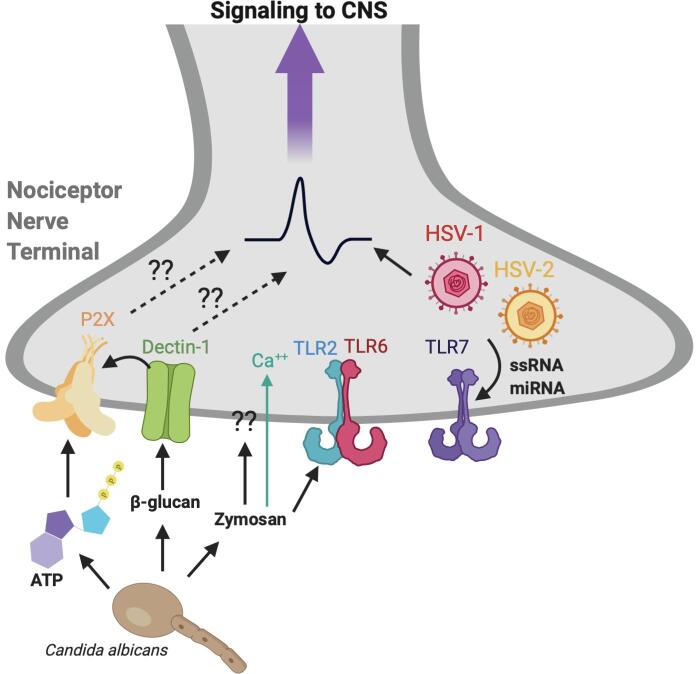
**Fungal and Viral PAMPs that cause nociception.** Candida albicans, an opportunistic fungal pathogen that causes painful candidiasis, can be sensed by nociceptors through their cell wall components, including Zymosan and β-glucan. Zymosans and β-glucan can be sensed by PRRs including Dectin-1 and TLR2 to activate nociceptors. Particularly, Zymosan binds TLR2 when it is in its dimeric form with TLR6. In cultured DRGs, Zymosan has been found to directly activate nociceptors through an unknown mechanism. C. albicans can also activate nociceptors through β-glucan induced dectin-1- TRP axis, and by ATP signaling through P2X3 receptor. ATP can also be released from C. albicans to bind non-peptidergic nociceptors, but it is unclear if this leads to pain behavior. Viral pathogens, including herpes simplex virus 1 (HSV-1) and herpes simplex virus 2 (HSV-2), may infect nociceptors directly to cause pain. Viral release of miRNA and ssRNA can also be sensed by TLR7 on nociceptors to cause neuronal activation. Image created with BioRender.com.

Viral infections are commonly associated with inflammation and pain. A major class of viral PAMPs are nucleic acids. Both viral genomes and viral replication products can trigger host responses and activation of the innate immune system (Sparrer and Gack, 2015). Viral pathogens have been found to release single stranded RNA and miRNAs which can be sensed by TLR7 on TRPA1+ neurons to cause pain and itch (Ji et al., 2016, Park et al., 2014) (Fig. 3). Further, viral pathogens have also been found to infect nociceptor neurons to cause pain. The most common viruses to do this are herpes simplex viruses 1 and 2 (HSV-1 and HSV-2) and varicella zoster virus (VZV) which leads to the painful skin rash called Shingles. Cutaneous HSV-1 infection in mice led to an increased mechanical allodynia (Silva et al., 2017). Post-herpetic neuralgia (PHN), which occurs following reactivation of VZV, leads to chronic neuropathic pain by leukocytes infiltrating DRGs, producing TNF and downregulating potassium channel Kir4.1 in satellite glial cells (Silva et al., 2017). A mouse leukemia virus (MuLV) has also been found to increase pain sensitivity, which was rescued in mice lacking an enzyme that degrades the amino acid tryptophan, indoleamine 2,3 dioxygenase (IDO) (Huang et al., 2016). The authors further found that acute influenza A infection increased pain sensitivity and increased IDO enzyme activity, but IDO1-decificent mice did not rescue hyperalgesia (Huang et al., 2016). Given the link between bacterial respiratory infections signaling to neurons to cause cough (Ruhl et al., 2020), it is also possible that viral respiratory illnesses may directly signal to, or infect nociceptors in the airways to cause cough and this is an important area for future research.

#### 1.2.7 Fungal pathogens and pain

Fungal opportunistic pathogens can cause painful infections in the skin and genitourinary tract. Fungal PAMPs include cell wall components such as chitin, β-glucans, and mannoproteins (Taghavi et al., 2017, Sorrell and Chen, 2009). Zymosan, a glucan made up of repeated glucose molecules, has been known to induce pain in animal models (Meller and Gebhart, 1997). One group found that zymosan induced mechanical hypernociception in a mouse model of arthritis, that was dependent on TLR2 and MyD88 induced cytokine and chemokine signaling (Guerrero et al., 2012).

*Candida albicans* is an opportunistic yeast pathogen and a common member of the human oral and gut microbiome. *Candida albicans* and other species in genus *Candida* can cause painful infections, commonly referred to as candidiasis (Turner and Butler, 2014). It has been found that TRPV1 + DRG neurons could directly detect *C. albicans* and cause the release of CGRP, which led to increased expression of IL-23 in CD301b+ dendritic cells to promote production of IL-17A and resistance to candidiasis (Kashem et al., 2015). The same group went on to show that cutaneous TRPV1+ neuronal activation promoted type 17 immune signaling, and this was sufficient for host defense against *C. albicans* and *S. aureus* infections (Cohen et al., 2019). However, these studies do not identify the molecular mechanism through which nociceptors sense *C. albicans.* Recently, a group found that *C. albicans* derived soluble β-glucan is able to bind Dectin-1-TRP channel axis on DRG neurons, leading to depolarization and release of CGRP (Maruyama et al., 2017). Dectin-1 is an NK-cell-receptor-like-C-type lectin that can act as a PRR by its ability to bind β-glucan carbohydrates and initiate innate immune signaling cascades (Brown, 2006). The authors also found that *C. albicans* can cause mechanical allodynia by β-glucan signaling through the Dectin-1 receptor in neurons which can induce adenosine triphosphate (ATP) influx though the vesicular nucleotide transporter (VNUT) on ketotinocytes, which can then be released to bind to neurons through P2X (Maruyama et al., 2018). Previously, it was found that nociceptor neurons express purinoceptors, such as P2X3 and P2X2/3, and ATP can bind to these receptors to cause neuroinflammation and neuropathic pain (Jarvis et al., 2002). Further, it has been described that P2X receptors are calcium permeable and play a role in many forms of chronic pain (Bernier et al., 2018) Figure 3 In a recent non-peer reviewed publication, a group found that *C. albicans* can secrete ATP which can directly activate non-peptidergic sensory neurons by binding to P2RX7, and this correlates with increased host-defense after infection (Edwards et al., 2020). However, more mechanistic work is needed to determine if the release of ATP by *C. albicans* can directly affect pain behavior, and in what context. The pathogenicity of *C. albicans* and other fungi has been extensively studied (Mayer et al., 2013). Yet, it is still unclear how and when these opportunistic pathogens are able to inflict pain on the host. More mechanistic work to elucidate the nature of how opportunistic pathogens interact with neurons may help us better design new approaches to treat painful infections in the genitourinary tract, skin and oral cavity.

Damage associated molecular patterns (DAMPs) also contribute significantly to pain signaling both during sterile tissue injury and following infection by microbial pathogens including by bacteria, viruses and fungi. DAMPs are molecules that alert the innate and adaptive immune system that an injury has occurred. DAMPs can be found in nuclear/cytosolic compartments, or can be extracellular matrix proteins that mediate their effects by binding to PRRs such as TLRs. DAMPs mediate pain by binding to receptors found on both sensory neurons and immune cells. DAMPs that mediate pain include endogenous molecules such as ATP, heat shock proteins (HSPs) or high-mobility group box 1 (HMGB1), which are released from necrotic and apoptotic cells as well as by other cells in response to microbial components or cellular stress. Nociceptor sensory neurons express P2X3 channels which detects ATP, and TLR4 which can detect HSP70 or HMGB1 (Kuang et al., 2012, van Zoelen et al., 2009, Burnstock, 2016, Luong et al., 2012). HMGB1 signaling has been found to drive neuroinflammation and pain through TLR4 (Andersson and Tracey, 2011, Scaffidi et al., 2002). HMGB1 is a ubiquitously expressed nuclear DNA-binding protein that regulates many important cellular processes including DNA replication, repair, and recombination. When a cell is under stress, HMGB1 can translocate to the cytosol and be secreted as a ligand that can bind PRRs including TLR2, TLR4, TLR9, CD24, RAGE, Siglec and Mac-1 (Kato and Svensson, 2015). Further, many studies have found that anti-HMGB1 antibodies or drugs that promote degradation of HMGB1 reduce pain following nerve injury, LPS administration and other forms of neuropathy (Kato and Svensson, 2015). More work is needed to further characterize the role of DAMPs in microbe-induced acute and chronic pain. It is likely that a combination of DAMPs and PAMPs contribute to pain signaling in the context of infection or dysbiosis. We recommend a chapter by Kato and Svensson for a larger overview of more DAMPs in pain signaling (Kato and Svensson, 2015).

Ultimately, we have seen that pathogenic microorganisms can directly communicate with sensory afferent neurons in multiple ways. Bacteria can produce and secrete toxins, *N*-formyl peptides, and other molecules that can bind to nociceptors to cause hyperalgesia or block pain. We also described how different microorganisms, including fungal and viral pathogens have their own molecular patterns that can be sensed by nociceptors to induce pain.

## 2 Direct interactions between symbiotic microbes and nociceptors

While the molecular mechanisms by which microbial pathogens induce nociceptor activation or modulate their activity are more well characterized, the role of specific gut symbionts and their products in regulating nociceptor neuron function and pain is a relatively new area of research. Many of the same molecules that pathogens harbor are also present in gut or skin-resident symbionts, including LPS, flagellin, peptidoglycan, lipoproteins, and fungal chitin, and these molecules have the potential to act directly on sensory afferents (Boller and Felix, 2009). Gut microbes or their products may also leak into tissues following an epithelial barrier breach to bind directly to nociceptors.

**Fig 4:**
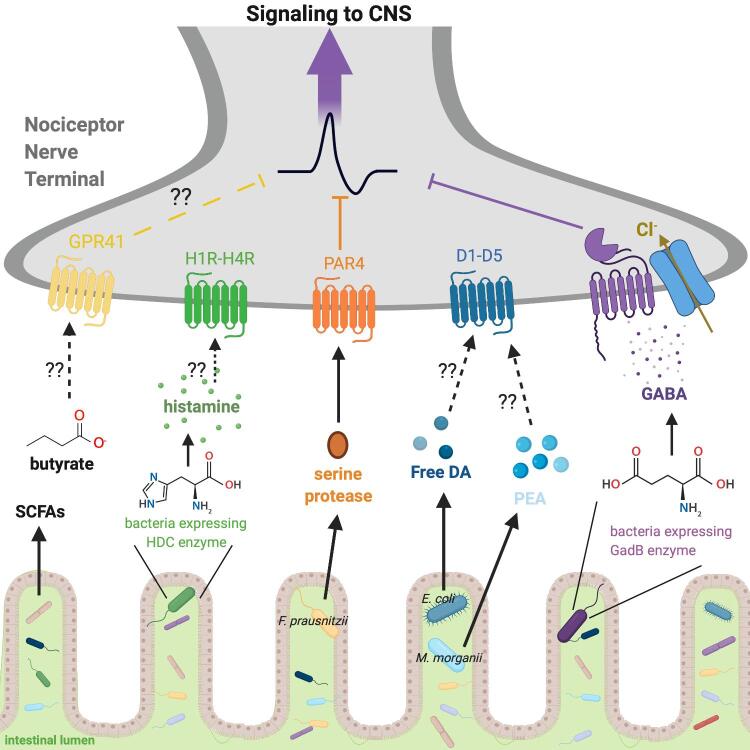
**Direct interactions between symbiotic microbes and a gut innervating sensory afferent neuron (nociceptor nerve terminal)**. Symbiontic bacteria  ferment foods high in  fiber to supply SCFAs to the host, including butyrate, propionate and acetate. Nociceptors have been found to express SCFA receptor GPR41/FFAR3 and butyrate has been suggested to have analgesic effects, but the mechanism has not been elucidated. Bacteria expressing the enzyme histidine decarboxylase (HDC) can catabolize histidine into histamine which may bind to receptors histamine receptors (H1R-H4R) on neurons, yet more work to understand which receptor is needed and if this signaling leads to pain. Bacteria such as *F. prausnitzii* can secrete serine proteases which can bind to protease activating receptor 4 (PAR4) to block activation of nociceptors. Other bacteria, such as *E. coli* and *M. Morganii* can process catechols in gut lumen to release free, bioactive dopamine (DA) and molecularly similar molecules, such as PEA. These may then bind nociceptors, but it is unknown their effect on pain perception. Addititonally, bacteria that express a gene for the enzyme glutamate decarboxylase β (GadB)  are able to catabolize glutamate into GABA which can then bind to GABA receptors on nociceptors leading to an influx of the anion, chloride, to hyperpolarize the membrane and block neurotransmission. Image created with BioRender.com.

As we described above, recent studies indicate a role for the gut microbiome in animal models of visceral, inflammatory, and neuropathic pain (Amaral et al., 2008, Luczynski et al., 2017, Shen, 2017). However, whether microbial signals directly act on nociceptor neurons in these pain models are not clear. Below, we propose known or plausible molecular mechanisms through which *gut* symbionts may communicate with gut-extrinsic sensory afferent neurons to affect pain. We will discuss how bacterial secreted biogenic amines and bioactive molecules including short-chain fatty acids (SCFAs), neurotransmitters such as catecholamines and GABA, and other molecules such as proteases and histamine may act on sensory neurons (Sudo, 2019) (Fig. 4). We note that some of these potential mechanisms of symbiotic microbe-neuron signaling have been identified in the context of other types of afferent neurons (e.g. sympathetic neurons), and therefore will require further investigation in the specific context of pain.

### 2.1 Microbial metabolites: SCFAs

Microbial metabolism is a vital aspect to the symbiotic relationship between microbes and host. Microbes contribute to the production of vital primary metabolites and secondary metabolites that the host can utilize to maintain health and homeostasis. An extensive review of microbial metabolites in health and disease can be found by Sharon et al. (2014). In the context of pain, microbial metabolites may interact with neurons directly or indirectly if their detection is relayed through gut epithelial cells including enterochromaffin cells, which we will outline later in this review. Here, we will address some known direct molecular mechanisms between microbial metabolites signal to sensory afferents and propose hypothetical pathways that may also lead to the perception of pain.

Gut microbes help humans digest foods high in fiber and provide necessary metabolites to the host. Gut microbes help the host digest soluble fibers, such as galacto-oligosaccharides and fructo-oligosaccharides, by fermenting them and releasing SCFAs such as butyrate, acetate and propionate (. SCFAs have been found to have beneficial effects on host metabolism and energy homeostasis and many aspects of host physiology (Koh et al., 2016, De Vadder and Kovatcheva-Datchary, 2014). Recently it was shown that prenatal exposure to SCFA is important for development of the autonomic nervous system and neurite outgrowth of tyrosine hydroxylase (TH) positive neurons that innervate cardiac tissue (Kimura et al., 2020). Supplementation of propionate to pups born from GF mothers rescues neural deficits and autonomic control of cardiac output (Kimura et al., 2020). SCFA bind G-protein coupled receptors such as GRP41/FFAR3, GRP43, GRP109A to initiate signaling cascades in immune cells, epithelial cells, adipose tissue and sensory afferents of the autonomic nervous system (Koh et al., 2016). The SCFA, propionate, has been found to bind GPR41/FFAR3 on sympathetic neurons of the superior cervical ganglion and can regulate sympathetic nervous system activity and energy homeostasis (Kimura et al., 2011). The SCFA receptor GPR41/FFAR3 is also expressed on spinal DRGs, trigeminal ganglion as well as sensory neurons of the vagus nerve (Nøhr et al., 2015) SCFAs have also been found to play an important role in visceral sensation (Dalile et al., 2019, van Thiel et al., 2020). The SCFA, butyrate, has been found to have analgesic properties in an acute model of visceral pain, yet the mechanism remains to be determined (Russo et al., 2016). It has been suggested that butyrate may regulate CGRP release to decrease visceral hyperalgesia (van Thiel et al., 2020, Gschossmann et al., 2001) but more mechanistic evidence is needed. It would be interesting to determine if intrinsic expression of SCFA receptors on DRG neurons is involved. Another group found that butyrate may promote visceral hypersensitivity by increasing nerve growth factor (NGF) secretion by enteric glial cells (Long et al., 2018) (. Given the opposing effects in these studies, it is possible that butyrate and other SCFA have multiple roles depending on the given situation in the host. SCFA have also been found to play a vital role in intestinal physiology as they help maintain epithelial cell integrity, immune cell function and hormone secretion (Parada Venegas et al., 2019, Priyadarshini et al., 2018). Given this, SCFA levels may determine what microbes or microbial products leak into the gut parenchyma to act on neurons and this may be another way SCFA control signaling to sensory afferent nociceptors.

### 2.2 Neurotransmitters: GABA and catecholamines

Gut microbes have been found to utilize and/or produce neurotransmitters such as catecholamines, γ-aminobutyric acid (GABA) and serotonin. We will discuss serotonin below in our indirect mechanism of microbial communication through epithelial cells to neurons. A review on neurotransmitter modulation by gut microbiota can be found by Strandwitz, P. (Strandwitz, 2018) . Here, we will discuss how microbes consume and produce GABA in the context of pain as well as catecholamines.

GABA is the primary inhibitory neurotransmitter. GABA acts by hyperpolarizing the pre-synaptic terminal of a neuron, through inducing an influx of chloride. This blocks a neuron from firing an action potential and releasing synaptic vesicles. GABAergic signaling has been linked to many neurologic disorders including depression/anxiety, and nociception (Bravo et al., 2011, Strandwitz et al., 2019). In fact, Gabapentin, a structural analog to GABA, has been used in the clinic for pain management to thermal and mechanical pain for decades (Rose et al., 2002). Bacteria have been found to produce and/or consume GABA and impact host neurological function (Strandwitz et al., 2019). In one study, researchers examined differences in the cerebral and plasma metabolome of GF mice and Ex-GF mice, and found significantly less GABA levels in plasma and luminal contents from GF mice (Matsumoto et al., 2013). Further, metagenomic studies have identified *Lactobacillus* and *Bifidobacterium* as being lower in patients with visceral hyperalgesia and irritable bowel syndrome (IBS) (Chong, 2019) Several *in vivo* studies have found that probiotics that include strains from the *Lactobacillus* and *Bifidobacterium* genera are beneficial in reducing visceral pain (Lomax et al., 2019). Both *Lactobacillus* and *Bifidobacterium* are genera that have been found to express the enzyme glutamate decarboxylase β (GadB), which decarboxylates glutamate into GABA (Barrett et al., 2012, Li et al., 2013) Fig. 4.

There have also been many human clinical trials examining the role of probiotics on IBS symptoms. In one of these studies, a randomized, double blind, controlled trial examined the effects of a probiotic cocktail vs. placebo on multiple IBS symptoms in 100 patients (Drouault-Holowacz et al., 2008). The authors examined visceral pain/discomfort, stool frequency and overall quality of life. They found that the patients who took the probiotic cocktail had significantly less pain after 4 weeks of treatment compared to placebo, but the probiotic cocktail did not make a significant difference on the overall score of IBS symptoms. A recent meta-analysis of studies examined the efficacy and safety of probiotics for IBS, and found that overall, single strain probiotics given for a short amount of time may be the most beneficial in mitigating IBS symptoms including pain (Li et al., 2020, Li et al., 2020). Yet, the authors could not draw a conclusion as to which specific probiotic was the most beneficial. This is most likely due to the heterogeneity of the microbiome from patient to patient and in humans from various parts of the world (Li et al., 2020). One interesting further direction of these studies would be to examine if any of the bacteria used in beneficial probiotics share any genes or functional pathways that may shed light on a universal mechanism of action of these probiotics. This may enable the generation of a synthetic probiotic that may work to lower pain without drastically changing the composition of the human gut flora.

In a recent *in vivo* animal study, Pokusaeva *et al*. examined GABA’s role on visceral pain sensitivity in mice by engineering a *Bifidobacterium* strain to express the GadB enzyme, which decarboxylates glutamate into GABA (Fig. 4). They found that mice colonized with *Bifidobacterium* + GadB have increased GABA production and reduced visceral pain sensation compared to mice colonized with the *Bifidobacterium* GadB deficient strain (Pokusaeva et al., 2017). This study highlights another therapeutic avenue for treating chronic pain disorders: by engineering bacteria to produce analgesic molecules that could be given as a probiotic (Zhao et al., 2016). More mechanistic work needs to be done to examine if other microbial species are capable of producing GABA or structurally similar molecules, and how this signaling may impact the development or attenuation of different forms of chronic pain. Of relevance, one study found that GABA blocks pathological pain but not acute TRPV1 pain signals (Hanack et al., 2015)

Recently, a group identified a novel lipoprotein that acts on the GABA receptor to inhibit pain. They found that administering *E. coli* Nissle 1917 decreased visceral pain associated with IBS (Perez-Berezo et al., 2017). Using biochemical purification and visceral pain studies, they found that Nissle produced an analgesic lipoprotein, C12AsnGABAOH, that is able to cross the epithelial barrier, bind to a GABA_B_ receptor, and inhibit neuronal calcium influx.

Catecholamines, including norepinephrine, epinephrine and dopamine are monoamine neurotransmitters that are secreted in response to activation of the sympathetic nervous system or the “Flight or Fight” response. Patients with chronic pain have been found to have increased sympathetic autonomic activity (Perry et al., 1989) with an increase in levels of circulating catecholamines and a decrease in catechol-O-methyl transferase (COMT), an enzyme that metabolizes catecholamines (Ciszek et al., 2016). Pathogens and symbionts have been found to use catecholamines for growth, and some pathogens have been found to use catecholamines to increase virulence (Strandwitz et al., 2019). It has been proposed that pathogenic microbes use adrenergic neurotransmitters for quorum sensing to regulate bacterial gene expression for their growth and survival (Sperandio et al., 2003). While it is not clear exactly how symbiotic microbes change in response to chronic sympathetic activation, it is possible this may cause symbionts to produce metabolites that may function like dopamine, or could turn on expression of genes that promote catecholamine activity. One study examined the difference in biologically active catecholamines in the intestinal lumen from specific-pathogen free (SPF) mice compared to GF mice and found GF mice have significantly less bioactive, unconjugated catecholamines than SPF (Asano et al., 2012). This group went on to show that inoculation of *E. coli* into GF mice increased levels of bioactive free catecholamine, but not when *E. coli* were deficient in a gene coding for a β-glucuronidase enzyme. They therefore suggested that inactive catecholamines in the gut lumen may be deconjugated by bacteria that express the β-glucuronidase enzyme which enables deconjugation of dopamine into a bioactive form (Asano et al., 2012). Additionally, in a recent screen of microbial metabolites that activate GPCRs, it was found that more than a dozen commensal supernatants are able to activate dopamine and histamine receptors (Chen et al., 2019). The authors went on to examine one strain, *M. morganii*, that has been previously reported to produce biogenic amines, including dopamine, and found that it can produce phenethylamine (PEA), a metabolite structurally related to dopamine and capable of binding to dopamine receptors (Chen et al., 2019). While these studies have not examined if dopamine or PEA bind to DRG neurons to modulate pain behavior, previous work has shown that DRG neurons and/or the dorsal horn express all 5 dopamine receptors (D1R-D5R) (Megat et al., 2018). Moreover, Megat *et al.* have found that DR1/DR5 antagonism transiently inhibits neuropathic pain. They also show this was sexually dimorphic, where D5R plays a more critical role in males and D1R plays a more critical role in females (Megat et al., 2018). While more work needs to be done to determine if microbially produced DA or structurally similar metabolites, such as DEA, can directly modulate pain, the work by Chen *et al.* emphasizes the importance of large *in vitro* screens for identifying microbially produced bioactive molecules, as well as for identifying precursors for these molecules such as amino acids.

### 2.3 Microbial proteases and histamine

Proteases are enzymes that catalyze the degradation of proteins into smaller peptides or amino acids. They are essential for life and found across every kingdom of life. Microbially produced proteases have been found to be important for drug development and there is a large industry for engineering bacteria to produce them (Razzaq et al., 2019). In mammals, proteases bind to protease-activated receptors (PARs), a family of GPCRs that include PAR1, PAR2, PAR3, and PAR4 (Heuberger and Schuepbach, 2019). Activation of these receptors has commonly been linked to chronic inflammatory diseases and pain (Heuberger and Schuepbach, 2019). One group found that PAR2 activation on colonic innervating DRG neurons leads to increased neuronal excitability, and found long term changes in membrane permeability after administering a PAR2 agonist (Kayssi et al., 2007). Further it was found that the TRPV1 channel on DRG neurons was sensitized by PAR2 in a PKC-dependent manner and this may contribute to sensation of pain (Dai et al., 2007). However, the PAR2 agonist has not been identified. Recently, it was found that a group of symbionts are able to reduce the excitability of DRG neurons *in vitro*, and this effect was blocked when DRGs were treated with a serine protease inhibitor (Sessenwein et al., 2017). They went on to show that a microbially produced serine protease was specifically binding to PAR4 on DRG neurons to block their excitability by increasing rheobase, or the membrane excitability potential. Further, Sessenwein *et al.* found inhibitors of cysteine proteases, acid proteases, metalloproteases, or aminopeptidases did not have the same effect as serine protease inhibitors in restoring DRG excitability (Sessenwein et al., 2017). Given the differing effects of neuronal excitation following PAR activation, more molecular studies investigating what agonists lead to these effects are needed. It is plausible that gut microbiota are responsible for producing proteases that are capable of directly binding to PARs on nociceptors, and engineering probiotics to target these pathways may be one potential therapeutic avenue for chronic pain. In addition to these direct interceptions, proteases can also be stored in mast cells and released upon mast cell degranulation to signal to neurons, which is discussed below in the “indirect signaling” section.

Microbes have also been found to play a role in histamine production. Histamine is a biogenic amine that is important for mucus and electrolyte secretion, peristalsis, response to allergens, and wakefulness (Bischoff and Krämer, 2007, Scammell et al., 2019, Barcik et al., 2016. Histamine is released in granules by mast cells, which we will discuss below in indirect mechanisms of pain through immune cells. Histamine can directly bind nociceptors through binding to a family of histamine receptor GPCRs, (H1R-H4R) (Smolinska et al., 2014, Chen et al., 2019). It has been extensively described that histamine is a key mediator of nociception through activation of H1R and H2R on neurons (Smith et al., 2007). Further, it has been found that blocking H3R and H4R enhances mechanical hyperalgesia in a mouse model of neuropathic pain, and increases the number of mast cells in the injured DRG (Smith et al., 2007). It has also been found that pre-exposure of histamine or serotonin to colonic sensory neurons can lead to an increase in expression of TRPV4 receptors, which leads to increased neuronal sensitization and could contribute to visceral hypersensitivity (Cenac et al., 2010). Histamine has been found to be higher in the small intestine of conventional mice compared to GF mice (Beaver and Wostmann, 1962). Upon further examination, microbes have been found to express a gene for the enzyme histidine decarboxylase (HDC), which enables them to catabolize histidine into histamine (Stratton et al., 1991) . In a recent screen of microbial metabolites that activate GPCRs, it was found that two bacteria, *M. morganii* and *L. reuteri*, are able to convert dietary histidine into histamine to increase gut motility (Chen et al., 2019). More work needs to be done to understand the role of microbially produced histamine in nociception and if it may activate receptors. Further, it would be interesting to examine whether microbially produced histamine may prime nociceptors to express TRP channels.

## 3 Summary

Microbes have many ways of directly communicating with sensory neurons either proximally or distally. It is possible that gut symbionts may be contributing to chronic pain through numerous ways at various times of the disease. It is also possible that other perturbations in the host, such as epithelial barrier dysfunction, may contribute to increased circulation of microbial products that may attenuate chronic pain and this may become more pathogenic. Many neurotransmitters addressed in this section have transporters that are expressed by gut epithelial cells to allow their transport into the intestinal lumen, however much more work is needed to elucidate these signaling pathways. In order to better understand the molecular mechanisms of direct microbial-neuron crosstalk, better models are needed to examine multiple potential ways microbes may be acting at the same time to alter neuronal physiology.

## 4 Indirect interactions between symbiotic microbes and sensory afferents in pain

The barrier surfaces including the gut are lined with epithelial cells and immune cells that crosstalk with sensory afferents. In addition to direct microbe-neuron crosstalk, these cells (epithelial and immune) are critical intermediators that are also sensing microbial products and relaying signals to the somatosensory nervous system through products including serotonin or other neurotransmitters. This is especially true at homeostasis where barrier dysfunction may not be occurring, and therefore, symbionts in the gut would be communicating via epithelial and immune cells to neurons. We appreciate that this is a major area of research and we cannot cover all of the potential indirect cellular mechanisms by which microbial signals are relayed from the microbiome to nociceptor afferents. In this section, we will outline potential indirect mechanisms through which symbiotic bacteria communicate with epithelial cells or innate immune cells which then signal to sensory afferents to mediate pain.

### 4.1 Microbial crosstalk between epithelial cells and neurons

Epithelial cells form a thin layer that act as a barrier between the microbiota and the parenchyma of many host tissues. The epithelial barrier is important in symbiotic relationship between microbe and host. In the gut, the epithelial layer is composed of many different types of cells that play a role in sensing their environment to mediate gut homeostasis, mucus secretion, and host defense. Further, some of the cells that make up the epithelial layer in the intestine, lungs and airways are capable of signaling to nearby neurons by secreting neurotransmitters, hormones and neuropeptides. Below, we will highlight one indirect mechanism of microbial signaling through enterochromaffin cells to nociceptor neurons.

#### 4.1.1 Enterochromaffin cells

Enterochromaffin cells (ECCs) are a subset of enteroendocrine cells in the gut that are important for chemosensation (Bellono et al., 2017) . Enterochromaffin cells are similar to neurons in that they are electrically excitable, and express voltage gated ion channels (Bellono et al., 2017). They also express specific receptors for signal transduction commonly found on neurons, including TRPA1, SCFA receptors, and an olfactory receptor 558 (Olfr558) (Bellono et al., 2017) While ECCs do not make up a large portion of cells in the intestinal epithelial layer (<1%), they are responsible for generating ~90% of the body’s serotonin (Berger et al., 2009). Serotonin is an important neurotransmitter that has been associated with healthy neurological function. Perturbed serotonin signaling has been found to be associated with mood and stress related disorders such as anxiety and depression (Berger et al., 2009). Serotonin is derived from tryptophan, an essential amino acid. Gut microbiota have been found to generate tryptophan from dietary proteins which can then be catabolized into many bioactive molecules, which may promote microbial fitness in the intestine (Fung et al., 2019). For serotonin synthesis, tryptophan is catabolized into tryptamine which binds to ECCs in the gut epithelial layer to induce the release of 5-hydroxytryptamine (5-HT, serotonin)(Roager and Licht, 2018) Microbes have been found to induce the release of serotonin from ECCs in a reversible manner (Yano et al., 2015). A review on microbially produced tryptophan in health and disease can be found by Roager HM, and Lincht TR (Roager and Licht, 2018)( Serotonin has also been found to play a role in visceral sensation and pain; however, the role of the microbially regulated serotonin in pain sustentation has not been fully elucidated (Lomax et al., 2019) ). Serotonin has been found to modulate visceral pain sensation through TRPV4 activation (Cenac et al., 2010). The authors found that histamine and serotonin can potentiate TRPV4 through multiple mechanisms: TRPV4 agonist-induced calcium signaling by phospholipase C and protein kinase C phosphorylation of TRPV4, translocating TRPV4 from the nuclear zone to the plasma membrane, and by phospholipase A2 fostering the potentiation of TRVP4 (Cenac et al., 2010). Additionally, bile acid metabolites were found to stimulate the release of serotonin from ECCs to activate DRG neurons, and this may play a role in visceral hyperalgesia (Yu et al., 2019), and as we will discuss, microbes play a role in metabolizing bile acids which may influence this signaling pathway as well. Given the link between visceral pain and depression and anxiety, as commonly seen in patients with IBS, intestinal serotonin signaling may underlie these behaviors. Additionally, the role of serotonin in visceral pain may be sexually dimorphic as estorgens may regulate serotonin reuptake and degradation (Paredes et al., 2019) However, it is unclear how much of a role microbial regulation of serotonin signaling plays in chronic pain diseases. It is possible that faulty tryptophan production by microbes, or lack of microbial species capable of facilitating ECC secretion of serotonin may contribute to increased visceral pain phenotypes. To conclude, we discussed how enterochromaffin cells are capable of indirect signaling between microbes to neurons via the release of serotonin. It is possible there are many other cells in the epithelial layer that facilitate crosstalk between microbes and neurons.

### 4.2 Microbial crosstalk between immune cells and neurons

Human babies receive their first exposure to microbes from their mothers during their exit from the womb along the vaginal tract. Microbes have been found to play a vital role in development of the immune system (Mazmanian et al., 2005). Gomez de Agüero *et al* showed that even during gestation, microbial colonization in the mother increases intestinal group 2 innate lymphoid cells in the pups and this helps them be better able to avoid an inflammatory response to pathogenic microbes postnatally (Gomez de Aguero et al., 2016). It has become increasing clear that microbial – immune – neural signaling is important for healthy neurological function. Nociceptor neurons express receptors for many products secreted by immune cells, such as cytokines, proteases, growth factors and lipids. An extensive review of how immune cells signal to neurons to initiate pain can be found by Pinho-Ribeiro et al. (2017). Further, microbes have been found to impact several immune pathways which are capable of signaling to the peripheral and central nervous system, and an extensive review can be found by Fung et al. (2017). Here we will discuss indirect signaling through which microbes signal to T regulatory cells, signaling to mast cells to release neuromodulators, and signaling to glial cells which can mediate neuronal physiology.

#### 4.2.1 T cells

Given the links between the microbiome and immune system development, the human gut microbiota has been extensively studied to identify immunomodulatory organisms (Geva-Zatorsky, 2017). Microbes have been found to regulate gene expression of the host, including gene expression of neurons ( Geva-Zatorsky, 2017, Yissachar et al., 2017). Recently, it was unexpectedly found that symbionts that are able to induce either Th17 or RORγ+ T-regulatory (Treg) cells *in vivo* are able to downregulate expression of many genes involved in synaptic transmission, regulation of neurotransmitter release, and neuropeptide signaling (Yissachar et al., 2017). Further, this group found that some microorganisms that can affect Treg differentiation, such as *C. ramosum* and *B. ovatus*, are also able to trigger DRG neuronal activation *in vitro* through calcium influx (Yissachar et al., 2017). Yet, the molecular pathways of how microbes activate these neurons have not been determined.

Extensive work to understand microbial neuroimmune signaling has been conducted using the commensal microbe *B. fragilis. B fragilis* has been found to secrete polysaccharide A (PSA) and SCFAs that can bind directly to TLR2 on Foxp3(+) Treg cells. This leads to the release of the anti-inflammatory cytokine IL-10 which can block neuroinflammation (Round et al., 2011). The same group went on to show that *B. fragilis* can be used as a probiotic to protect from neuroinflammation in an Experimental Autoimmune Encephalomyelitis (EAE) animal model of multiple sclerosis, and this may be a potential therapeutic avenue for painful demyelinating diseases (Ochoa-Repáraz et al., 2009); Wang et al., 2014). Further, microbial metabolites, such as SCFAs, have been found to increase Treg differentiation and IL-10 production to block neuroinflammation (Haghikia et al., 2015). Intrathecal injections of IL-10 have profound analgesic effects in neuroinflammatory pain models (Milligan et al., 2012). More work is needed to examine how microbes may impact Treg mediated IL-10 release to modulate nociceptors, and to examine if other commensal bacteria are capable of having the same effect. Another review on neuro-immune interactions at barrier surfaces can be found by Viega-Fernandes H, and Mucida D (Veiga-Fernandes and Mucida, 2016)

### 4.3 Mast cells

Mast cells are regulatory cells found to play an important role in gut homeostasis and innate immunity. They are located at mucosal surfaces and play an important role in host defense. Mast cells secrete mediators that are stored in granules, a process called degranulation. Some of these mediators are histamine, proteases, NGF and ATP which can act as neuromodulators both in the peripheral and central nervous system (Héron and Dubayle, 2013, Koroleva et al., 2019). When mast cells recognize MAMPs and/or PAMPs by TLRs, they can release their granules to initiate a signaling cascade leading to activation of the adaptive immune system. Recently, there is emerging evidence that one avenue of microbe-host communication may be mast cells, and this is of particular interest in allergic inflammation and fibromyalgesia (Forsythe, 2016, Theoharides et al., 2019. A thorough review on mast cells, bacteria and intestinal immunity can be found by Bischoff and Krämer (2007). Below we will outline two pathways by which microbes first signal to mast cells which then release mediators that can act on neurons.

One mediator that mast cells can secrete are proteases, which can bind to their receptor, PARs. As we outlined above, neurons express PARs which may activate or inactivate nociceptor neurons. Mast cells can secrete proteases that can activate TRPA1 + DRG neurons through binding to PAR2, and this has been found to cause inflammatory pain (Dai et al., 2007). Further, upon infection or stress, mast cells can signal to colonocytes to release tryptase, a serine protease that can cleave and activate PARs to regulate tight junction assembly in the gut epithelium (Jacob et al., 2005). Perturbation of this system may allow for microbial products leaking into the parenchyma and signaling to neurons.

Bile acids are synthesized from cholesterol in the liver and released into the small intestine to help aid the digestion and absorption of fat and fat-soluble vitamins. Commensal microbes have been found to play a role in bile acid synthesis (Brestoff and Artis, 2013) as well as the metabolism of primary bile acid into primary and secondary metabolites (Lefebvre et al., 2009, Wahlström et al., 2016). Bile acids play a role in visceral pain hypersensitivity by communicating with mast cells. It has been found that one primary bile acid metabolite, deoxycholic acid (DCA), is able to activate vagal and spinal DRG neurons in the distal colon through binding to farnesoid X receptor and a bile acid GPCR (GPBAR1/ TGR5) on mast cells (Li et al., 2019). After DCA binds, it induces the release of NGF from mucosal mast cells to activate the TrkA/ TRPV1 axis on gut innervating nociceptors to increase visceral hypersensitivity (Li et al., 2019).

An important area of future research is to examine the role of the microbial signaling to mast cells to cause release of mediators that have been shown to bind to and activate nociceptor neurons. Furthermore, more work is needed to examine the role of potential microbial- mast cell signaling for the regulation of the epithelial barrier and how this regulation may go awry in chronic pain disorders.

#### 4.3.1 Microglia, and glial cells

In the brain and spinal cord, glial cells, including microglia and astrocytes, play a critical role in regulating immune function. In the peripheral nervous system (DRGs, trigeminal ganglia, and sympathetic and parasympathetic ganglia) satellite glial cells (SGC) play a critical role in maintaining sensory neuron function. SGC have been found to respond to acute noxious stimuli and signal to neurons via ATP release and P2X7 receptor activation (Lemes et al., 2018). Macrophages also infiltrate DRGs and nerves following injury to imitate inflammation and pain and may be a target for therpaeutic intervention (Ohashi et al., 2015). A comprehensive review on how glia-neuro interactions underlie chronic pain can be found by Ji et al. (2013). However, there have been relatively few studies done to elucidate the role of microbial - glial - neuronal signaling in the context of pain. Here, we will briefly describe bacterial communication with microglia and how this may lead to pain. A recent review on microbial signaling to microglia in the context of chronic pain can be found by Dworsky-Fried et al. (2020).

Bacterial components, such as peptidoglycan and lipopolysaccharide have been found to bind PRRs such as TLR2 and TLR4 on microglia and astrocytes in the spinal cord. This initiates a signaling cascade leading to the release of TNF-α and IL-1β which can then bind nociceptors and lead to onset of inflammatory pain (Ji et al., 2016). Microbes have also been found to alter the development, function and maturation of the microglia by impacting their gene expression and morphology (Erny et al., 2015, Fung et al., 2017). In these studies, it was also found that microglia deficient for the SCFA receptor FFAR2 phenocopy microglia from GF mice, suggesting that microbial metabolites are important for healthy glial function (Erny et al., 2015). Further, microglia have been implicated in pathogenesis and attenuation of pain after an injury, as seen by their increased numbers and activated morphology (Denk et al., 2016). Recently, it was found that microglia from GF mice cannot stimulate viral immunity or protect from viral induced neuronal demyelination (Brown et al., 2019). It would be interesting to examine whether microglia from GF mice are able to respond to injury. Overall, these studies emphasize that microbial communication to microglia is important for nervous system mediated host-response and development of an inflammatory response. While the molecular mechanisms of are not well understood, it is possible that microbial priming of microglia may play a vital role in response to injury and development of chronic pain. Other glial types, such as enteric glia may also communicate with sensory afferent neurons, including the DRG and facilitate communicating signals from gut microbes. Advancement in transcriptomic techniques like single cell sequencing may help elucidate different populations of glial cells in the DRG and the peripheral nervous system at large that may respond to microbial cues to alter pain sensation.

## 5 Summary and future directions

Microorganisms have many ways of interacting with sensory afferent neurons through direct and indirect communication, which leads to modulation of pain behaviors and host defense. Pathogens can secrete bacterial toxins, *N*-formyl peptides, and other molecules that are capable of binding to nociceptors. Further, there are many PAMPs in bacteria, fungi and viruses that can bind directly to and activate nociceptors to affect pain. Symbiotic microbes can also signal to neurons, either directly through secretion of metabolites, neurotransmitters and other molecules, or indirectly through first interacting with epithelial cells or immune cells. In some cases, microbial signaling can lead to a decrease in neuronal excitability and analgesic behavior, while in the other cases pathogenic signaling can lead to increased neuronal excitability and increased pain perception. Understanding the transitions between beneficial and pathogenic behavior will be vital to decipher the host-microbe relationship. Given the complexity and heterogeneity of the microbial ecosystem from person to person, underlying universal molecular mechanisms contributing to pain have been hard to identify. An added layer of complexity lies within the specific pain perception that can be generated: inflammatory, neuropathic, or visceral pain all manifest differently and could be thought of as different diseases. Another important question is with regards to the sexual dimorphism of pain perception and whether the gut microbiota may contribute to these differences through affecting sex hormonal signaling.

Each signaling pathway discussed above needs thorough molecular studies to better understand *when* symbionts signal to neurons, whether a change in microbiota happens before onset of pain perception or helps the attenuation of it after a prior stressor, and how the host adapts to lingering/repeated inputs. There is also evidance that neuropeptides produced by the enteric nervous system may affect microbial composition and that this communication may be bidirectional (Aresti Sanz and El Aidy, 2019). As highlighted in a review by Guo *et al*, clinical and *in vivo* animal studies have confounding reports for probiotics or antibiotics being beneficial in the treatment of visceral, inflammatory and neuropathic pain. This is likely due to the timing in which either probiotics or antibiotics were administered relative to pain onset (Guo et al., 2019). Moreover, future studies should put emphasis on stratification of microorganisms and the nuances within a microbial community. Previous work from the immunology field has shown that microbial strains from the same species modulate the immune system differently (Geva-Zatorsky, 2017) and this may also apply to modulation of DRG neurons and pain perception.

Because chronic pain disorders are so complex and often are a manifestation of genetics, early life stress, and a myriad of other environmental triggers, the models we have right now are not the most equipped to tackle these complicated diseases. We may need to reevaluate how we are asking related questions and use a compilation of models from the neuroscience, microbiology, and immunology fields. Recent studied have shown that the gut microbiome can regulate gene expression of many neuronal cell types, including the DRG, enteric neurons, sympatheric neurons and microglia in the bain (Muller et al., 2020, Obata et al., 2020, Chu et al., 2019). A deeper look into how microbes regulate expression of neurons within the DRG is warranted to examine how microbes may prime pain behavior on a transcriptional level. Eventually, a lucid understanding into the molecular mechanisms behind microbe-neuron communication may help us design better therapies for diseases of chronic pain including migraines, chemotherapy induced neuropathy, diabetic neuropathy, and IBS.

## CRediT authorship contribution statement

**Valentina N. Lagomarsino:** Writing - original draft, Visualization. **Aleksandar D. Kostic:** Writing - review & editing, Supervision. **Isaac M. Chiu:** Writing - review & editing, Supervision.

## Declaration of Competing Interest

I.M.C: receives sponsored research support from GSK and Allergan Pharmaceuticals. I.MC. is a member of scientific advisory boards for GSK and Kintai pharmaceuticals.

A.D.K. is a co-founder of and holds equity in DeepBiome Therapeutics and FitBiomics.

## References

[b0005] Abraira V., Ginty D. (2013). The sensory neurons of touch. Neuron.

[b0010] Amaral F.A., Sachs D., Costa V.V., Fagundes C.T., Cisalpino D., Cunha T.M., Ferreira S.H., Cunha F.Q., Silva T.A., Nicoli J.R., Vieira L.Q., Souza D.G., Teixeira M.M. (2008). Commensal microbiota is fundamental for the development of inflammatory pain. Proc. Natl. Acad. Sci..

[b0015] Andersson U., Tracey K.J. (2011). HMGB1 Is a Therapeutic Target for Sterile Inflammation and Infection. Annu. Rev. Immunol..

[b0020] Aresti Sanz J., El Aidy S. (2019). Microbiota and gut neuropeptides: a dual action of antimicrobial activity and neuroimmune response. Psychopharmacology.

[b0025] Asano Y., Hiramoto T., Nishino R., Aiba Y., Kimura T., Yoshihara K., Koga Y., Sudo N. (2012). Critical role of gut microbiota in the production of biologically active, free catecholamines in the gut lumen of mice. Am. J. Physiol.-Gastrointest. Liver Physiol..

[b0030] Baral P., Udit S., Chiu I.M. (2019). Pain and immunity: implications for host defence. Nat. Rev. Immunol..

[b0035] Barcik W., Pugin B., Westermann P., Perez N.R., Ferstl R., Wawrzyniak M., Smolinska S., Jutel M., Hessel E.M., Michalovich D., Akdis C.A., Frei R., O'Mahony L. (2016). Histamine-secreting microbes are increased in the gut of adult asthma patients. J. Allergy Clin. Immunol..

[b0040] Barrett E., Ross R.P., O'Toole P.W., Fitzgerald G.F., Stanton C. (2012). γ-Aminobutyric acid production by culturable bacteria from the human intestine. J. Appl. Microbiol..

[b0045] Basbaum A.I., Bautista D.M., Scherrer G., Julius D. (2009). Cellular and Molecular Mechanisms of Pain. Cell.

[b0050] Beaver, M.H., Wostmann, B.S., 1962. Histamine and 5-hydroxytryptamine in the intestinal tract of germ-free animals, animals harbouring one microbial species and conventional ANIMALS. Br. J. Pharmacol. Chemother., 19(3), 385–393. https://doi.org/10.1111/j.1476-5381.1962.tb01443.x. 10.1111/j.1476-5381.1962.tb01443.x PMC1482218 13970018

[b0055] Bellono, N. W., Bayrer, J. R., Leitch, D.B., Castro, J., Zhang, C., O’Donnell, T. A., Brierley, S. M., Ingraham, H. A., Julius, D., 2017. Enterochromaffin Cells Are Gut Chemosensors that Couple to Sensory Neural Pathways. Cell, 170(1), 185–198.e16. https://doi.org/10.1016/j.cell.2017.05.034. 10.1016/j.cell.2017.05.034 PMC5839326 28648659

[b0060] Benini, A., DeLeo, J.A., 1999. René Descartes’ Physiology of Pain: Spine, 24(20), 2115. https://doi.org/10.1097/00007632-199910150-00010. 10.1097/00007632-199910150-00010 10543009

[b0065] Berger M., Gray J.A., Roth B.L. (2009). The expanded biology of serotonin. Annu. Rev. Med..

[b0070] Bernier L.-P., Ase A.R., Séguéla P. (2018). P2X receptor channels in chronic pain pathways: P2X receptors in chronic pain. Br. J. Pharmacol..

[b0075] Bischoff, S.C., Krämer, S., 2007. Human mast cells, bacteria, and intestinal immunity. Immunol. Rev., 217(1), 329–337. https://doi.org/10.1111/j.1600-065X.2007.00523.x. 10.1111/j.1600-065X.2007.00523.x 17498069

[b0080] Blake K.J., Baral P., Voisin T., Lubkin A., Pinho-Ribeiro F.A., Adams K.L., Roberson D.P., Ma Y.C., Otto M., Woolf C.J., Torres V.J., Chiu I.M. (2018). Staphylococcus aureus produces pain through pore-forming toxins and neuronal TRPV1 that is silenced by QX-314. Nat. Commun..

[b0085] Boller T., Felix G. (2009). A renaissance of elicitors: perception of microbe-associated molecular patterns and danger signals by pattern-recognition receptors. Annu. Rev. Plant Biol..

[b0095] Braniste, V., Al-Asmakh, M., Kowal, C., Anuar, F., Abbaspour, A., Toth, M., Korecka, A., Bakocevic, N., Ng, L. G., Kundu, P., Gulyas, B., Halldin, C., Hultenby, K., Nilsson, H., Hebert, H., Volpe, B. T., Diamond, B., & Pettersson, S. (2014). The gut microbiota influences blood-brain barrier permeability in mice. Sci. Transl. Med., 6(263), 263ra158-263ra158. https://doi.org/10.1126/scitranslmed.3009759. 10.1126/scitranslmed.3009759 PMC4396848 25411471

[b0100] Bravo J.A., Forsythe P., Chew M.V., Escaravage E., Savignac H.M., Dinan T.G., Bienenstock J., Cryan J.F. (2011). Ingestion of Lactobacillus strain regulates emotional behavior and central GABA receptor expression in a mouse via the vagus nerve. Proc. Natl. Acad. Sci..

[b0105] Brestoff J.R., Artis D. (2013). Commensal bacteria at the interface of host metabolism and the immune system. Nat. Immunol..

[b0110] Brown, D.G., Soto, R., Yandamuri, S., Stone, C., Dickey, L., Gomes-Neto, J.C., Pastuzyn, E.D., Bell, R., Petersen, C., Buhrke, K., Fujinami, R.S., O’Connell, R.M., Stephens, W.Z., Shepherd, J.D., Lane, T.E., Round, J.L., 2019. The microbiota protects from viral-induced neurologic damage through microglia-intrinsic TLR signaling. ELife, 8, e47117. https://doi.org/10.7554/eLife.47117. 10.7554/eLife.47117 PMC6634972 31309928

[b0115] Brown G.D. (2006). Dectin-1: a signalling non-TLR pattern-recognition receptor. Nat. Rev. Immunol..

[b0120] Bullman S., Meyerson M., Kostic A.D. (2017). Emerging concepts and technologies for the discovery of microorganisms involved in human disease. Annu. Rev. Pathol. Mech. Dis..

[b0125] Burnstock, G., 2016. Purinergic mechanisms and pain. In: Advances in Pharmacology (Vol. 75, pp. 91–137). Elsevier. https://doi.org/10.1016/bs.apha.2015.09.001. 10.1016/bs.apha.2015.09.001 26920010

[b0130] Cenac N., Altier C., Motta J.-P., d'Aldebert E., Galeano S., Zamponi G.W., Vergnolle N. (2010). Potentiation of TRPV4 signalling by histamine and serotonin: an important mechanism for visceral hypersensitivity. Gut.

[b0135] Chen H., Nwe P.-K., Yang Y.i., Rosen C.E., Bielecka A.A., Kuchroo M., Cline G.W., Kruse A.C., Ring A.M., Crawford J.M., Palm N.W. (2019). A forward chemical genetic screen reveals gut microbiota metabolites that modulate host physiology. Cell.

[b0140] Chiu, I.M., 2018. Infection, Pain, and Itch. Neuroscience Bulletin, 34(1), 109–119. https://doi.org/10.1007/s12264-017-0098-1. 10.1007/s12264-017-0098-1 PMC5799131 28144843

[b0145] Chiu I.M., Heesters B.A., Ghasemlou N., Von Hehn C.A., Zhao F., Tran J., Wainger B., Strominger A., Muralidharan S., Horswill A.R., Wardenburg J.B., Hwang S.W., Carroll M.C., Woolf C.J. (2013). Bacteria activate sensory neurons that modulate pain and inflammation. Nature.

[b0155] Chiu I.M., von Hehn C.A., Woolf C.J. (2012). Neurogenic inflammation and the peripheral nervous system in host defense and immunopathology. Nat. Neurosci..

[bib913] Chong Pei Pei, Yong (2019). The Microbiome and Irritable Bowel Syndrome – A Review on the Pathophysiology, Current Research and Future Therapy. Front. Microbiol..

[b0165] Chu C., Murdock M.H., Jing D., Won T.H., Chung H., Kressel A.M., Tsaava T., Addorisio M.E., Putzel G.G., Zhou L., Bessman N.J., Yang R., Moriyama S., Parkhurst C.N., Li A., Meyer H.C., Teng F., Chavan S.S., Tracey K.J., Regev A., Schroeder F.C., Lee F.S., Liston C., Artis D. (2019). The microbiota regulate neuronal function and fear extinction learning. Nature.

[b0170] Ciszek B.P., O’Buckley S.C., Nackley A.G. (2016). Persistent catechol-O-methyltransferase–dependent pain is initiated by peripheral β-adrenergic receptors. Anesthesiology.

[b0175] Cohen J.A., Edwards T.N., Liu A.W., Hirai T., Jones M.R., Wu J., Li Y., Zhang S., Ho J., Davis B.M., Albers K.M., Kaplan D.H. (2019). Cutaneous TRPV1+ neurons trigger protective innate type 17 anticipatory immunity. Cell.

[b0180] Costello E.K., Lauber C.L., Hamady M., Fierer N., Gordon J.I., Knight R. (2009). Bacterial community variation in human body habitats across space and time. Science.

[b0185] Costigan M., Scholz J., Woolf C.J. (2009). Neuropathic pain: a maladaptive response of the nervous system to damage. Annu. Rev. Neurosci..

[b0190] Cryan J.F., Dinan T.G. (2012). Mind-altering microorganisms: the impact of the gut microbiota on brain and behaviour. Nat. Rev. Neurosci..

[b0195] Cryan J.F., O'Riordan K.J., Cowan C.S.M., Sandhu K.V., Bastiaanssen T.F.S., Boehme M., Codagnone M.G., Cussotto S., Fulling C., Golubeva A.V., Guzzetta K.E., Jaggar M., Long-Smith C.M., Lyte J.M., Martin J.A., Molinero-Perez A., Moloney G., Morelli E., Morillas E., O'Connor R., Cruz-Pereira J.S., Peterson V.L., Rea K., Ritz N.L., Sherwin E., Spichak S., Teichman E.M., van de Wouw M., Ventura-Silva A.P., Wallace-Fitzsimons S.E., Hyland N., Clarke G., Dinan T.G. (2019). The microbiota-gut-brain axis. Physiol. Rev..

[b0200] Dai Y.i., Wang S., Tominaga M., Yamamoto S., Fukuoka T., Higashi T., Kobayashi K., Obata K., Yamanaka H., Noguchi K. (2007). Sensitization of TRPA1 by PAR2 contributes to the sensation of inflammatory pain. J. Clin. Invest..

[b0205] Dalile, B., Van Oudenhove, L., Vervliet, B., Verbeke, K., 2019. The role of short-chain fatty acids in microbiota–gut–brain communication. Nat. Rev. Gastroenterol. Hepatol., 16(8), 461–478. https://doi.org/10.1038/s41575-019-0157-3. 10.1038/s41575-019-0157-3 31123355

[bib911] De Vadder Filipe, Kovatcheva-Datchary Petia, Gilles Mithieux (2014). Microbiota-Generated Metabolites Promote Metabolic Benefits via Gut-Brain Neural Circuits. Cell.

[b0210] Defaye M., Gervason S., Altier C., Berthon J.-Y., Ardid D., Filaire E., Carvalho F.A. (2020). Microbiota: a novel regulator of pain. J. Neural Transm..

[b0215] Denk, F., Crow, M., Didangelos, A., Lopes, D.M., McMahon, S.B., 2016. Persistent alterations in microglial enhancers in a model of chronic pain. Cell Reports, 15(8), 1771–1781. https://doi.org/10.1016/j.celrep.2016.04.063. 10.1016/j.celrep.2016.04.063 27184839

[b0220] Dopkins N., Nagarkatti P.S., Nagarkatti M. (2018). The role of gut microbiome and associated metabolome in the regulation of neuroinflammation in multiple sclerosis and its implications in attenuating chronic inflammation in other inflammatory and autoimmune disorders. Immunology.

[b0225] Drouault-Holowacz S, Bieuvelet S, Burckel A, Cazaubiel M, Dray X, Marteau P., 2008. A double blind randomized controlled trial of a probiotic combination in 100 patients with irritable bowel syndrome. Gastroenterol. Clin. Biol. 2008 Feb;32(2):147-52. doi: 10.1016/j.gcb.2007.06.001. Epub 2008 Mar 4. PMID: 18387426. 10.1016/j.gcb.2007.06.001 18387426

[b0230] Dubin A.E., Patapoutian A. (2010). Nociceptors: the sensors of the pain pathway. J. Clin. Invest..

[b0235] Dworsky-Fried Z., Kerr B.J., Taylor A.M.W. (2020). Microbes, microglia, and pain. Neurobiol. Pain.

[b0240] Edwards T.N., Zhang S., Liu A., Cohen J.A., Zhou P.Y., Mogavero S., Hube B., Berman J., Bougnoux M.-E., Mathers A.R., Gaffen S.L., Albers K.M., Koerber H.R., Davis B.M., D’Enfert C., Kaplan D.H. (2020). Extracellular ATP released from Candida albicans activates non-peptidergic neurons to augment host defense. Immunology.

[b0245] Erny D., Hrabě de Angelis A.L., Jaitin D., Wieghofer P., Staszewski O., David E., Keren-Shaul H., Mahlakoiv T., Jakobshagen K., Buch T., Schwierzeck V., Utermöhlen O., Chun E., Garrett W.S., McCoy K.D., Diefenbach A., Staeheli P., Stecher B., Amit I., Prinz M. (2015). Host microbiota constantly control maturation and function of microglia in the CNS. Nat. Neurosci..

[b0250] Forsythe P. (2016). Microbes taming mast cells: implications for allergic inflammation and beyond. Eur. J. Pharmacol..

[b0255] Foster J.A., Rinaman L., Cryan J.F. (2017). Stress & the gut-brain axis: regulation by the microbiome. Neurobiol. Stress.

[b0260] Foster K.R., Schluter J., Coyte K.Z., Rakoff-Nahoum S. (2017). The evolution of the host microbiome as an ecosystem on a leash. Nature.

[b0265] Fung T.C., Olson C.A., Hsiao E.Y. (2017). Interactions between the microbiota, immune and nervous systems in health and disease. Nat. Neurosci..

[b0270] Fung T.C., Vuong H.E., Luna C.D.G., Pronovost G.N., Aleksandrova A.A., Riley N.G., Vavilina A., McGinn J., Rendon T., Forrest L.R., Hsiao E.Y. (2019). Intestinal serotonin and fluoxetine exposure modulate bacterial colonization in the gut. Nat. Microbiol..

[b0275] Furness, J.B., Stebbing, M.J., 2018. The first brain: Species comparisons and evolutionary implications for the enteric and central nervous systems. Neurogastroenterol. Motility, 30(2), e13234. https://doi.org/10.1111/nmo.13234. 10.1111/nmo.13234 29024273

[b0280] Gilbert, J.A., 2015. Social behavior and the microbiome. ELife, 4, e07322. https://doi.org/10.7554/eLife.07322. 10.7554/eLife.07322 PMC4379491 25826451

[b0290] Grundy, D., 2002. Neuroanatomy of visceral nociception: Vagal and splanchnic afferent. Gut, 51(Supplement 1), i2–i5. https://doi.org/10.1136/gut.51.suppl_1.i2. 10.1136/gut.51.suppl_1.i2 PMC1867719 12077054

[bib914] Geva-Zatorsky Naama, Denis Kasper (2017). Mining the Human Gut Microbiota for Immunomodulatory Organisms. Cell.

[b0285] Gomez de Aguero M., Ganal-Vonarburg S.C., Fuhrer T., Rupp S., Uchimura Y., Li H., Steinert A., Heikenwalder M., Hapfelmeier S., Sauer U., McCoy K.D., Macpherson A.J. (2016). The maternal microbiota drives early postnatal innate immune development. Science.

[b0295] Gschossmann J.M., Coutinho S.V., Miller J.C., Huebel K., Naliboff B., Wong H.C., Walsh J.H., Mayer E.A. (2001). Involvement of spinal calcitonin gene-related peptide in the development of acute visceral hyperalgesia in the rat. Neurogastroenterol. Motil..

[b0300] Guerrero A.T.G., Cunha T.M., Verri W.A., Gazzinelli R.T., Teixeira M.M., Cunha F.Q., Ferreira S.H. (2012). Toll-like receptor 2/MyD88 signaling mediates zymosan-induced joint hypernociception in mice: participation of TNF-α, IL-1β and CXCL1/KC. Eur. J. Pharmacol..

[b0305] Guo R., Chen L.-H., Xing C., Liu T. (2019). Pain regulation by gut microbiota: molecular mechanisms and therapeutic potential. Br. J. Anaesth..

[b0310] Haghikia A., Jörg S., Duscha A., Berg J., Manzel A., Waschbisch A., Hammer A., Lee D.-H., May C., Wilck N., Balogh A., Ostermann A., Schebb N.H., Akkad D., Grohme D., Kleinewietfeld M., Kempa S., Thöne J., Demir S., Müller D., Gold R., Linker R. (2015). Dietary fatty acids directly impact central nervous system autoimmunity via the small intestine. Immunity.

[b0315] Hanack C., Moroni M., Lima W., Wende H., Kirchner M., Adelfinger L., Schrenk-Siemens K., Tappe-Theodor A., Wetzel C., Kuich P.H., Gassmann M., Roggenkamp D., Bettler B., Lewin G., Selbach M., Siemens J. (2015). GABA blocks pathological but not acute TRPV1 pain signals. Cell.

[b0320] Hayashi, F., Smith, K.D., Ozinsky, A., Hawn, T.R., Yi, E.C., Goodlett, D.R., Eng, J.K., Akira, S., 2001. The innate immune response to bacterial ¯agellin is mediated by Toll-like receptor 5. 410, 5. 10.1038/35074106 11323673

[b0325] Helley M.P., Abate W., Jackson S.K., Bennett J.H., Thompson S.W.N. (2015). The expression of Toll-like receptor 4, 7 and co-receptors in neurochemical sub-populations of rat trigeminal ganglion sensory neurons. Neuroscience.

[b0330] Héron A., Dubayle D. (2013). A focus on mast cells and pain. J. Neuroimmunol..

[b0335] Heuberger D.M., Schuepbach R.A. (2019). Protease-activated receptors (PARs): Mechanisms of action and potential therapeutic modulators in PAR-driven inflammatory diseases. Thrombosis J..

[b0340] Hoban A.E., Stilling R.M., Moloney G., Shanahan F., Dinan T.G., Clarke G., Cryan J.F. (2018). The microbiome regulates amygdala-dependent fear recall. Mol. Psychiatry.

[b0345] Hsiao E., McBride S., Hsien S., Sharon G., Hyde E., McCue T., Codelli J., Chow J., Reisman S., Petrosino J., Patterson P., Mazmanian S. (2013). Microbiota modulate behavioral and physiological abnormalities associated with neurodevelopmental disorders. Cell.

[b0350] Hu G., Huang K., Hu Y., Du G., Xue Z., Zhu X., Fan G. (2016). Single-cell RNA-seq reveals distinct injury responses in different types of DRG sensory neurons. Sci. Rep..

[b0360] Huang, L., Ou, R., Rabelo de Souza, G., Cunha, T.M., Lemos, H., Mohamed, E., Li, L., Pacholczyk, G., Randall, J., Munn, D.H., Mellor, A.L., 2016. Virus infections incite pain hypersensitivity by inducing indoleamine 2,3 dioxygenase. PLOS Pathogens, 12(5), e1005615. https://doi.org/10.1371/journal.ppat.1005615. 10.1371/journal.ppat.1005615 PMC4863962 27168185

[b0365] Jacob C., Yang P.-C., Darmoul D., Amadesi S., Saito T., Cottrell G.S., Coelho A.-M., Singh P., Grady E.F., Perdue M., Bunnett N.W. (2005). Mast cell tryptase controls paracellular permeability of the intestine: role of protease-activated receptor 2 and β-arrestins. J. Biol. Chem..

[b0370] Jarvis M.F., Burgard E.C., McGaraughty S., Honore P., Lynch K., Brennan T.J., Subieta A., van Biesen T., Cartmell J., Bianchi B., Niforatos W., Kage K., Yu H., Mikusa J., Wismer C.T., Zhu C.Z., Chu K., Lee C.-H., Stewart A.O., Polakowski J., Cox B.F., Kowaluk E., Williams M., Sullivan J., Faltynek C. (2002). A-317491, a novel potent and selective non-nucleotide antagonist of P2X3 and P2X2/3 receptors, reduces chronic inflammatory and neuropathic pain in the rat. Proc. Natl. Acad. Sci..

[b0375] Ji R.-R., Chamessian A., Zhang Y.-Q. (2016). Pain regulation by non-neuronal cells and inflammation. Science.

[b0380] Ji R.-R., Berta T., Nedergaard M. (2013). Glia and pain: Is chronic pain a gliopathy?:. Pain.

[b0385] Julius D. (2013). TRP channels and pain. Annu. Rev. Cell Dev. Biol..

[b0390] Kashem S., Riedl M., Yao C., Honda C., Vulchanova L., Kaplan D. (2015). Nociceptive sensory fibers drive interleukin-23 production from CD301b+ dermal dendritic cells and drive protective cutaneous immunity. Immunity.

[b0395] Kato, J., Svensson, C.I., 2015. Role of Extracellular Damage-Associated Molecular Pattern Molecules (DAMPs) as Mediators of Persistent Pain. In Progress in Molecular Biology and Translational Science (Vol. 131, pp. 251–279). Elsevier. https://doi.org/10.1016/bs.pmbts.2014.11.014. 10.1016/bs.pmbts.2014.11.014 25744676

[b0400] Kayssi A., Amadesi S., Bautista F., Bunnett N.W., Vanner S. (2007). Mechanisms of protease-activated receptor 2-evoked hyperexcitability of nociceptive neurons innervating the mouse colon: PAR _2_ activation evokes hyperexcitability of colonic DRG neurons. J. Physiol..

[b0405] Kimura I., Inoue D., Maeda T., Hara T., Ichimura A., Miyauchi S., Kobayashi M., Hirasawa A., Tsujimoto G. (2011). Short-chain fatty acids and ketones directly regulate sympathetic nervous system via G protein-coupled receptor 41 (GPR41). Proc. Natl. Acad. Sci..

[b0410] Kimura I., Miyamoto J., Ohue-Kitano R., Watanabe K., Yamada T., Onuki M., Aoki R., Isobe Y., Kashihara D., Inoue D., Inaba A., Takamura Y., Taira S., Kumaki S., Watanabe M., Ito M., Nakagawa F., Irie J., Kakuta H., Shinohara M., Iwatsuki K., Tsujimoto G., Ohno H., Arita M., Itoh H., Hase K. (2020). Maternal gut microbiota in pregnancy influences offspring metabolic phenotype in mice. Science.

[b0415] Koh A., De Vadder F., Kovatcheva-Datchary P., Bäckhed F. (2016). From dietary fiber to host physiology: short-chain fatty acids as key bacterial metabolites. Cell.

[b0420] Koroleva K., Gafurov O., Guselnikova V., Nurkhametova D., Giniatullina R., Sitdikova G., Mattila O.S., Lindsberg P.J., Malm T.M., Giniatullin R. (2019). Meningeal mast cells contribute to ATP-induced nociceptive firing in trigeminal nerve terminals: direct and indirect purinergic mechanisms triggering migraine pain. Front. Cell. Neurosci..

[b0425] Kuang X., Huang Y., Gu H.-F., Zu X.-y., Zou W.-Y., Song Z.-B., Guo Q.-L. (2012). Effects of intrathecal epigallocatechin gallate, an inhibitor of Toll-like receptor 4, on chronic neuropathic pain in rats. Eur. J. Pharmacol..

[b0430] Kumpitsch C., Koskinen K., Schöpf V., Moissl-Eichinger C. (2019). The microbiome of the upper respiratory tract in health and disease. BMC Biol..

[b0440] Lefebvre P., Cariou B., Lien F., Kuipers F., Staels B. (2009). Role of bile acids and bile acid receptors in metabolic regulation. Physiol. Rev..

[b0445] Lemes J.B.P., de Campos Lima T., Santos D.O., Neves A.F., de Oliveira F.S., Parada C.A., da Cruz Lotufo C.M. (2018). Participation of satellite glial cells of the dorsal root ganglia in acute nociception. Neurosci. Lett..

[b0450] Li B, Liang L, Deng H, Guo J, Shu H, Zhang L. Efficacy and safety of probiotics in irritable bowel syndrome: a systematic review and meta-analysis. Front Pharmacol. 2020 Apr 3;11:332. doi: 10.3389/fphar.2020.00332. PMID: 32317962; PMCID: PMC7147251. 10.3389/fphar.2020.00332 PMC7147251 32317962

[b0455] Li H., Li W., Liu X., Cao Y. (2013). *gadA* gene locus in *Lactobacillus brevis* NCL912 and its expression during fed-batch fermentation. FEMS Microbiol. Lett..

[b0460] Li, S., Hua, D., Wang, Q., Yang, L., Wang, X., Luo, A., Yang, C., 2020. The role of bacteria and its derived metabolites in chronic pain and depression: recent findings and research progress. Int. J. Neuropsychopharmacol., 23(1), 26–41. https://doi.org/10.1093/ijnp/pyz061. 10.1093/ijnp/pyz061 PMC7064053 31760425

[b0465] Li, W.-T., Luo, Q.-Q., Wang, B., Chen, X., Yan, X.-J., Qiu, H.-Y., & Chen, S.-L. (2019). Bile acids induce visceral hypersensitivity via mucosal mast cell–to–nociceptor signaling that involves the farnesoid X receptor/nerve growth factor/transient receptor potential vanilloid 1 axis. FASEB J., 33(2), 2435–2450. https://doi.org/10.1096/fj.201800935RR. 10.1096/fj.201800935RR 30260705

[b0470] Lister K.C., Bouchard S.M., Markova T., Aternali A., Denecli P., Pimentel S.D., Majeed M., Austin J.-S., Williams A.C. de C., Mogil J.S. (2020). Chronic pain produces hypervigilance to predator odor in mice. Curr. Biol..

[b0480] Liu, T., Berta, T., Xu, Z.-Z., Park, C.-K., Zhang, L., Lü, N., Liu, Q., Liu, Y., Gao, Y.-J., Liu, Y.-C., Ma, Q., Dong, X., Ji, R.-R., 2012a. TLR3 deficiency impairs spinal cord synaptic transmission, central sensitization, and pruritus in mice. J. Clin. Investig, 122(6), 2195–2207. https://doi.org/10.1172/JCI45414. 10.1172/JCI45414 PMC3366391 22565312

[b0485] Liu T., Gao Y.-J., Ji R.-R. (2012). Emerging role of Toll-like receptors in the control of pain and itch. Neurosci. Bull..

[b0495] Lomax A.E., Pradhananga S., Sessenwein J.L., O’Malley D. (2019). Bacterial modulation of visceral sensation: mediators and mechanisms. Am. J. Physiol.-Gastrointestinal Liver Physiol..

[b0500] Long, X., Li, M., Li, L.-X., Sun, Y.-Y., Zhang, W.-X., Zhao, D.-Y., & Li, Y.-Q. (2018). Butyrate promotes visceral hypersensitivity in an IBS-like model via enteric glial cell-derived nerve growth factor. Neurogastroenterol. Motility, 30(4), e13227. https://doi.org/10.1111/nmo.13227. 10.1111/nmo.13227 29052293

[b0505] Luczynski, P., Tramullas, M., Viola, M., Shanahan, F., Clarke, G., O’Mahony, S., Dinan, T.G., Cryan, J.F., 2017. Microbiota regulates visceral pain in the mouse. ELife, 6, e25887. https://doi.org/10.7554/eLife.25887. 10.7554/eLife.25887 PMC5478269 28629511

[b0510] Luong M., Zhang Y., Chamberlain T., Zhou T., Wright J.F., Dower K., Hall J.P. (2012). Stimulation of TLR4 by recombinant HSP70 requires structural integrity of the HSP70 protein itself. J. Inflamm..

[b0515] Ma B., Forney L.J., Ravel J. (2012). Vaginal microbiome: rethinking health and disease. Annu. Rev. Microbiol..

[b0520] Marion, E., Song, O.-R., Christophe, T., Babonneau, J., Fenistein, D., Eyer, J., Letournel, F., Henrion, D., Clere, N., Paille, V., Guérineau, N. C., Saint André, J.-P., Gersbach, P., Altmann, K.-H., Stinear, T. P., Comoglio, Y., Sandoz, G., Preisser, L., Delneste, Y., Brodin, P., 2014. Mycobacterial toxin induces analgesia in buruli ulcer by targeting the angiotensin pathways. Cell, 157(7), 1565–1576. https://doi.org/10.1016/j.cell.2014.04.040. 10.1016/j.cell.2014.04.040 24949969

[b0525] Maruyama K., Takayama Y., Kondo T., Ishibashi K.-I., Sahoo B.R., Kanemaru H., Kumagai Y., Martino M.M., Tanaka H., Ohno N., Iwakura Y., Takemura N., Tominaga M., Akira S. (2017). Nociceptors boost the resolution of fungal osteoinflammation via the TRP channel-CGRP-Jdp2 Axis. Cell Reports.

[b0530] Maruyama K., Takayama Y., Sugisawa E., Yamanoi Y.u., Yokawa T., Kondo T., Ishibashi K.-I., Sahoo B.R., Takemura N., Mori Y., Kanemaru H., Kumagai Y., Martino M.M., Yoshioka Y., Nishijo H., Tanaka H., Sasaki A., Ohno N., Iwakura Y., Moriyama Y., Nomura M., Akira S., Tominaga M. (2018). The ATP transporter VNUT mediates induction of dectin-1-triggered candida nociception. iScience.

[b0535] Matsumoto M., Kibe R., Ooga T., Aiba Y., Sawaki E., Koga Y., Benno Y. (2013). Cerebral low-molecular metabolites influenced by intestinal microbiota: a pilot study. Front. Syst. Neurosci..

[b0540] Mayer F.L., Wilson D., Hube B. (2013). *Candida albicans* pathogenicity mechanisms. Virulence.

[b0545] Mazmanian S.K., Liu C.H., Tzianabos A.O., Kasper D.L. (2005). An Immunomodulatory molecule of symbiotic bacteria directs maturation of the host immune system. Cell.

[b0550] McFall-Ngai M.J. (2014). The importance of microbes in animal development: lessons from the squid-vibrio symbiosis. Annu. Rev. Microbiol..

[b0555] Megat S., Shiers S., Moy J.K., Barragan-Iglesias P., Pradhan G., Seal R.P., Dussor G., Price T.J. (2018). A critical role for dopamine D5 receptors in pain chronicity in male mice. J. Neurosci..

[b0560] Meller S.T., Gebhart G.F. (1997). Intraplantar zymosan as a reliable, quantifiable model of thermal and mechanical hyperalgesia in the rat. Eur. J. Pain.

[b0565] Meseguer V., Alpizar Y.A., Luis E., Tajada S., Denlinger B., Fajardo O., Manenschijn J.-A., Fernández-Peña C., Talavera A., Kichko T., Navia B., Sánchez A., Señarís R., Reeh P., Pérez-García M.T., López-López J.R., Voets T., Belmonte C., Talavera K., Viana F. (2014). TRPA1 channels mediate acute neurogenic inflammation and pain produced by bacterial endotoxins. Nat. Commun..

[b0570] Milligan E.D., Penzkover K.R., Soderquist R.G., Mahoney M.J. (2012). Spinal Interleukin-10 therapy to treat peripheral neuropathic pain: INTRATHECAL IL-10 THERAPY. Neuromodul.: Technol. Neural Interface.

[b0575] Minter M.R., Zhang C., Leone V., Ringus D.L., Zhang X., Oyler-Castrillo P., Musch M.W., Liao F., Ward J.F., Holtzman D.M., Chang E.B., Tanzi R.E., Sisodia S.S. (2016). Antibiotic-induced perturbations in gut microbial diversity influences neuro-inflammation and amyloidosis in a murine model of Alzheimer’s disease. Sci. Rep..

[b0580] Moayedi M., Davis K.D. (2013). Theories of pain: from specificity to gate control. J. Neurophysiol..

[b0585] Muller P.A., Schneeberger M., Matheis F., Wang P., Kerner Z., Ilanges A., Pellegrino K., del Mármol J., Castro T.B.R., Furuichi M., Perkins M., Han W., Rao A., Pickard A.J., Cross J.R., Honda K., de Araujo I., Mucida D. (2020). Microbiota modulate sympathetic neurons via a gut–brain circuit. Nature.

[b0590] Nøhr M.K., Egerod K.L., Christiansen S.H., Gille A., Offermanns S., Schwartz T.W., Møller M. (2015). Expression of the short chain fatty acid receptor GPR41/FFAR3 in autonomic and somatic sensory ganglia. Neuroscience.

[b0595] Obata Y., Castaño Á., Boeing S., Bon-Frauches A.C., Fung C., Fallesen T., de Agüero M.G., Yilmaz B., Lopes R., Huseynova A., Horswell S., Maradana M.R., Boesmans W., Vanden Berghe P., Murray A.J., Stockinger B., Macpherson A.J., Pachnis V. (2020). Neuronal programming by microbiota regulates intestinal physiology. Nature.

[b0600] Ochoa-Cortes F., Ramos-Lomas T., Miranda-Morales M., Spreadbury I., Ibeakanma C., Barajas-Lopez C., Vanner S. (2010). Bacterial cell products signal to mouse colonic nociceptive dorsal root ganglia neurons. Am. J. Physiol.-Gastrointest. Liver Physiol..

[b0605] Ochoa-Repáraz J., Mielcarz D.W., Ditrio L.E., Burroughs A.R., Foureau D.M., Haque-Begum S., Kasper L.H. (2009). Role of gut commensal microflora in the development of experimental autoimmune encephalomyelitis. J. Immunol..

[b0610] Ohashi W., Hattori K., Hattori Y. (2015). Control of macrophage dynamics as a potential therapeutic approach for clinical disorders involving chronic inflammation. J. Pharmacol. Exp. Ther..

[b0615] Parada Venegas D., De la Fuente M.K., Landskron G., González M.J., Quera R., Dijkstra G., Harmsen H.J.M., Faber K.N., Hermoso M.A. (2019). Short chain fatty acids (SCFAs)-mediated gut epithelial and immune regulation and its relevance for inflammatory bowel diseases. Front. Immunol..

[b0620] Paredes S., Cantillo S., Candido K.D., Knezevic N.N. (2019). An association of serotonin with pain disorders and its modulation by estrogens. Int. J. Mol. Sci..

[b0625] Park C.-K., Xu Z.-Z., Berta T., Han Q., Chen G., Liu X.-J., Ji R.-R. (2014). Extracellular MicroRNAs activate nociceptor neurons to elicit pain via TLR7 and TRPA1. Neuron.

[b0630] Patapoutian A., Tate S., Woolf C.J. (2009). Transient receptor potential channels: targeting pain at the source. Nat. Rev. Drug Discov..

[b0635] Pavlov V.A., Tracey K.J. (2017). Neural regulation of immunity: molecular mechanisms and clinical translation. Nat. Neurosci..

[b0640] Pérez-Berezo T., Pujo J., Martin P., Le Faouder P., Galano J.-M., Guy A., Knauf C., Tabet J.C., Tronnet S., Barreau F., Heuillet M., Dietrich G., Bertrand-Michel J., Durand T., Oswald E., Cenac N. (2017). Identification of an analgesic lipopeptide produced by the probiotic Escherichia coli strain Nissle 1917. Nat. Commun..

[b0645] Perry F., Heller P.H., Kamiya J., Levine J.D. (1989). Altered autonomic function in patients with arthritis or with chronic myofascial pain:. Pain.

[b0655] Pinho-Ribeiro F.A., Baddal B., Haarsma R., O’Seaghdha M., Yang N.J., Blake K.J., Portley M., Verri W.A., Dale J.B., Wessels M.R., Chiu I.M. (2018). Blocking neuronal signaling to immune cells treats streptococcal invasive infection. Cell.

[b0660] Pinho-Ribeiro F.A., Verri W.A., Chiu I.M. (2017). Nociceptor sensory neuron–immune interactions in pain and inflammation. Trends Immunol..

[b0665] Pokusaeva K., Johnson C., Luk B., Uribe G., Fu Y., Oezguen N., Matsunami R.K., Lugo M., Major A., Mori-Akiyama Y., Hollister E.B., Dann S.M., Shi X.Z., Engler D.A., Savidge T., Versalovic J. (2017). GABA-producing *Bifidobacterium dentium* modulates visceral sensitivity in the intestine. Neurogastroenterol. Motil..

[b0670] Priyadarshini, M., Kotlo, K.U., Dudeja, P.K., Layden, B.T., 2018. Role of short chain fatty acid receptors in intestinal physiology and pathophysiology. In: D. M. Pollock (Ed.), Comprehensive Physiology (pp. 1091–1115). John Wiley & Sons, Inc. https://doi.org/10.1002/cphy.c170050. 10.1002/cphy.c170050 PMC6058973 29978895

[b0675] Qi J., Buzas K., Fan H., Cohen J.I., Wang K., Mont E., Klinman D., Oppenheim J.J., Howard O.M.Z. (2011). Painful pathways induced by TLR stimulation of dorsal root ganglion neurons. J. Immunol..

[b0680] Rakoff-Nahoum S., Medzhitov R. (2008). Innate immune recognition of the indigenous microbial flora. Mucosal Immunol..

[b0685] Razzaq A., Shamsi S., Ali A., Ali Q., Sajjad M., Malik A., Ashraf M. (2019). Microbial proteases applications. Front. Bioeng. Biotechnol..

[b0690] Roager H.M., Licht T.R. (2018). Microbial tryptophan catabolites in health and disease. Nat. Commun..

[b0695] Rose M.A., Kam P.C.A. (2002). Gabapentin: pharmacology and its use in pain management: Gabapentin. Anaesthesia.

[b0705] Round J.L., Lee S.M., Li J., Tran G., Jabri B., Chatila T.A., Mazmanian S.K. (2011). The toll-like receptor 2 pathway establishes colonization by a commensal of the human microbiota. Science.

[b0710] Ruhl C.R., Pasko B.L., Khan H.S., Kindt L.M., Stamm C.E., Franco L.H., Hsia C.C., Zhou M., Davis C.R., Qin T., Gautron L., Burton M.D., Mejia G.L., Naik D.K., Dussor G., Price T.J., Shiloh M.U. (2020). Mycobacterium tuberculosis sulfolipid-1 activates nociceptive neurons and induces cough. Cell.

[b0715] Russo R., De Caro C., Avagliano C., Cristiano C., La Rana G., Mattace Raso G., Berni Canani R., Meli R., Calignano A. (2016). Sodium butyrate and its synthetic amide derivative modulate nociceptive behaviors in mice. Pharmacol. Res..

[b0720] Sampson T.R., Debelius J.W., Thron T., Janssen S., Shastri G.G., Ilhan Z.E., Challis C., Schretter C.E., Rocha S., Gradinaru V., Chesselet M.-F., Keshavarzian A., Shannon K.M., Krajmalnik-Brown R., Wittung-Stafshede P., Knight R., Mazmanian S.K. (2016). Gut microbiota regulate motor deficits and neuroinflammation in a model of Parkinson’s Disease. Cell.

[b0725] Scaffidi, P., Misteli, T., Bianchi, M.E., 2010. Release of chromatin protein HMGB1 by necrotic cells triggers inflammation. Nature. 2002 Jul 11;418(6894):191-5. doi: 10.1038/nature00858. Erratum in: Nature. 2010 Sep 30;467(7315):622. PMID: 12110890. 10.1038/nature00858 12110890

[b0730] Scammell, T.E., Jackson, A.C., Franks, N.P., Wisden, W., Dauvilliers, Y., 2019. Histamine: Neural circuits and new medications. Sleep, 42(1). https://doi.org/10.1093/sleep/zsy183. 10.1093/sleep/zsy183 PMC6335869 30239935

[b0735] Schommer, N.N., Gallo, R.L., 2013. Structure and function of the human skin microbiome. Trends Microbiol., 21(12), 660–668. https://doi.org/10.1016/j.tim.2013.10.001. 10.1016/j.tim.2013.10.001 PMC4744460 24238601

[b0740] Sessenwein J.L., Baker C.C., Pradhananga S., Maitland M.E., Petrof E.O., Allen-Vercoe E., Noordhof C., Reed D.E., Vanner S.J., Lomax A.E. (2017). Protease-mediated suppression of DRG neuron excitability by commensal bacteria. J. Neurosci..

[b0745] Sharma N., Flaherty K., Lezgiyeva K., Wagner D.E., Klein A.M., Ginty D.D. (2020). The emergence of transcriptional identity in somatosensory neurons. Nature.

[b0750] Sharon G., Garg N., Debelius J., Knight R., Dorrestein P., Mazmanian S. (2014). Specialized metabolites from the microbiome in health and disease. Cell Metab..

[b0755] Sharon G., Sampson T.R., Geschwind D.H., Mazmanian S.K. (2016). The central nervous system and the gut microbiome. Cell.

[bib915] Shen, Mao (2017). Gut microbiota is critical for the induction of chemotherapy- induced pain. Nature Neuroscience.

[b0760] Silva J.R., Lopes A.H., Talbot J., Cecilio N.T., Rossato M.F., Silva R.L., Souza G.R., Silva C.R., Lucas G., Fonseca B.A., Arruda E., Alves-Filho J.C., Cunha F.Q., Cunha T.M. (2017). Neuroimmune–glia interactions in the sensory ganglia account for the development of acute herpetic neuralgia. J. Neurosci..

[b0765] Smith F.M., Haskelberg H., Tracey D.J., Moalem-Taylor G. (2007). Role of histamine H_3_ and H_4_ receptors in mechanical hyperalgesia following peripheral nerve injury. NeuroImmunoModulation.

[b0770] Smolinska S., Jutel M., Crameri R., O'Mahony L. (2014). Histamine and gut mucosal immune regulation. Allergy.

[b0775] Sorrell, T.C., Chen, S.C.A., 2009. Fungal-Derived Immune Modulating Molecules. In P. G. Fallon (Ed.), Pathogen-Derived Immunomodulatory Molecules (Vol. 666, pp. 108–120). Springer New York. https://doi.org/10.1007/978-1-4419-1601-3_9. 10.1007/978-1-4419-1601-3_9 20054979

[b0780] Sparrer K.MJ., Gack M.U. (2015). Intracellular detection of viral nucleic acids. Curr. Opin. Microbiol..

[b0785] Sperandio V., Torres A.G., Jarvis B., Nataro J.P., Kaper J.B. (2003). Bacteria-host communication: the language of hormones. Proc. Natl. Acad. Sci..

[b0790] Strandwitz P. (2018). Neurotransmitter modulation by the gut microbiota. Brain Res..

[b0795] Strandwitz P., Kim K.H., Terekhova D., Liu J.K., Sharma A., Levering J., McDonald D., Dietrich D., Ramadhar T.R., Lekbua A., Mroue N., Liston C., Stewart E.J., Dubin M.J., Zengler K., Knight R., Gilbert J.A., Clardy J., Lewis K. (2019). GABA-modulating bacteria of the human gut microbiota. Nat. Microbiol..

[b0800] Stratton J.E., Hutkins R.W., Taylor S.L. (1991). Biogenic amines in cheese and other fermented foods: a review. J. Food Prot..

[b0805] Sudo N. (2019). Biogenic amines: signals between commensal microbiota and gut physiology. Front. Endocrinol..

[b0810] Taghavi M., Khosravi A., Mortaz E., Nikaein D., Athari S.S. (2017). Role of pathogen-associated molecular patterns (PAMPS) in immune responses to fungal infections. Eur. J. Pharmacol..

[b0815] The Human Microbiome Project Consortium (2012). Structure, function and diversity of the healthy human microbiome. Nature.

[b0820] Theoharides T.C., Tsilioni I., Bawazeer M. (2019). Mast Cells, neuroinflammation and pain in fibromyalgia syndrome. Front. Cell. Neurosci..

[b0825] Turner, S.A., Butler, G., 2014. The Candida Pathogenic Species Complex. Cold Spring Harbor Perspectives in Medicine, 4(9), a019778–a019778. https://doi.org/10.1101/cshperspect.a019778. 10.1101/cshperspect.a019778 PMC4143104 25183855

[b0835] van Thiel I.A.M., Botschuijver S., de Jonge W.J., Seppen J. (2020). Painful interactions: Microbial compounds and visceral pain. Biochim. Biophys. Acta (BBA) – Mol. Basis Dis..

[b0840] van Zoelen M.A.D., Yang H., Florquin S., Meijers J.C.M., Akira S., Arnold B., Nawroth P.P., Bierhaus A., Tracey K.J., Poll T.V.D. (2009). Role of toll-like receptors 2 and 4, and the receptor for advanced glycation end products in high-mobility group box 1-induced inflammation in vivo:. Shock.

[b0845] Veiga-Fernandes H., Mucida D. (2016). Neuro-immune interactions at barrier surfaces. Cell.

[b0850] Vuong H.E., Yano J.M., Fung T.C., Hsiao E.Y. (2017). The microbiome and host behavior. Annu. Rev. Neurosci..

[b0855] Wadachi R., Hargreaves K.M. (2006). Trigeminal nociceptors express TLR-4 and CD14: a mechanism for pain due to infection. J. Dent. Res..

[b0860] Wade W.G. (2013). The oral microbiome in health and disease. Pharmacol. Res..

[b0865] Wahlström A., Sayin S., Marschall H.-U., Bäckhed F. (2016). Intestinal crosstalk between bile acids and microbiota and its impact on host metabolism. Cell Metab..

[b0870] Wang Y., Telesford K.M., Ochoa-Repáraz J., Haque-Begum S., Christy M., Kasper E.J., Wang L., Wu Y., Robson S.C., Kasper D.L., Kasper L.H. (2014). An intestinal commensal symbiosis factor controls neuroinflammation via TLR2-mediated CD39 signalling. Nat. Commun..

[b0875] Xu Z.-Z., Kim Y.H., Bang S., Zhang Y.i., Berta T., Wang F., Oh S.B., Ji R.-R. (2015). Inhibition of mechanical allodynia in neuropathic pain by TLR5-mediated A-fiber blockade. Nat. Med..

[b0880] Yang N.J., Chiu I.M. (2017). Bacterial signaling to the nervous system through toxins and metabolites. J. Mol. Biol..

[b0885] Yano J., Yu K., Donaldson G., Shastri G., Ann P., Ma L., Nagler C., Ismagilov R., Mazmanian S., Hsiao E. (2015). Indigenous bacteria from the gut microbiota regulate host serotonin biosynthesis. Cell.

[b0890] Yissachar N., Zhou Y., Ung L., Lai N.Y., Mohan J.F., Ehrlicher A., Weitz D.A., Kasper D.L., Chiu I.M., Mathis D., Benoist C. (2017). An intestinal organ culture system uncovers a role for the nervous system in microbe-immune crosstalk. Cell.

[b0895] Yu Y., Villalobos-Hernandez E.C., Pradhananga S., Baker C.C., Keating C., Grundy D., Lomax A.E., Reed D.E. (2019). Deoxycholic acid activates colonic afferent nerves via 5-HT _3_ receptor-dependent and -independent mechanisms. Am. J. Physiol.-Gastrointestinal Liver Physiol..

[b0905] Zhao A., Hu X., Li Y., Chen C., Wang X. (2016). Extracellular expression of glutamate decarboxylase B in Escherichia coli to improve gamma-aminobutyric acid production. AMB Express.

[b0910] Zheng P., Zeng B., Zhou C., Liu M., Fang Z., Xu X., Zeng L., Chen J., Fan S., Du X., Zhang X., Yang D., Yang Y., Meng H., Li W., Melgiri N.D., Licinio J., Wei H., Xie P. (2016). Gut microbiome remodeling induces depressive-like behaviors through a pathway mediated by the host’s metabolism. Mol. Psychiatry.

